# ﻿Order Euryalida (Echinodermata, Ophiuroidea), new species and new records from the South China Sea and the Northwest Pacific seamounts

**DOI:** 10.3897/zookeys.1090.76292

**Published:** 2022-03-30

**Authors:** Hasitha Nethupul, Sabine Stöhr, Haibin Zhang

**Affiliations:** 1 Institute of Deep-sea Science and Engineering, Chinese Academy of Sciences, CAS, 57200 Sanya, China Institute of Deep-Sea Science and Engineering, Chinese Academy of Sciences Sanya China; 2 University of Chinese Academy of Sciences, Beijing 100039, China University of Chinese Academy of Sciences Beijing China; 3 Swedish Museum of Natural History, Dept of Zoology, Box 50007, 10405 Stockholm, Sweden Swedish Museum of Natural History Stockholm Sweden

**Keywords:** *Asteroschema*, COI, molecular phylogeny, morphology, SEM, taxonomy

## Abstract

Ophiuroids were collected by the manned submersible ‘Shenhaiyongshi’ from the deep-sea seamounts in the South China Sea and Northwest Pacific regions at 602–1920 m depth, during 2018 to 2020. A total of nine species was identified, including two new species and seven new records from the South China Sea and one new record from the Northwest Pacific region. Two new species are described as *Asteroschemashenhaiyongshii***sp. nov.** and *Asteroschemadomogranulatum***sp. nov**. The seven new records included five species from the genus *Asteroschema*, and one species each from the genera *Asterostegus* and *Astrodendrum*. Comprehensive descriptions of morphological features are provided, including characteristics of the arm skeleton, as well as a phylogenetic analysis based on 16S and COI sequences. Intraspecific genetic distance ranges of Euryalida species from the present study were 0.34% to 1.38%, which was relatively low compared to other orders in the class Ophiuroidea. The present study suggests a high probability that species of the order Euryalida are more widely spread around the Indo-Pacific region than previously expected.

## ﻿Introduction

The order Euryalida Lamarck, 1816 (basket stars and snake stars) includes the families Euryalidae Gray, 1840, Asteronychidae Ljungman, 1867, and Gorgonocephalidae Ljungman, 1867, and these include the species with largest known body size in the class Ophiuroidea, the brittle stars (Stöhr et al. 2021). The majority of the Euryalida are epizoic, living attached to hosts, such as corals, gorgonians, and sponges (Baker 1980). Currently, the order Euryalida contains 193 accepted species within 48 genera (Stöhr et al. 2021). The largest families are Euryalidae and Gorgonocephalidae with 95% of all Euryalida (Stöhr et al. 2021). These two families include 44 genera (Euryalidae 11 genera, Gorgonocephalidae 33 genera).

This study presents species in the genera *Asteroschema* Örsted & Lütken in Lütken, 1856 and *Asterostegus* Mortensen, 1933 from Euryalidae, and *Astrodendrum* (Döderlein, 1902) from family Gorgonocephalidae, found in the South China Sea and on Northwest Pacific seamounts.

*Asteroschema* is one of the largest genera in the Euryalidae, but it is still ill-defined due to limited published information and high morphological similarity between the species. Currently, 33 species are included in *Asteroschema* and the most recently described one was *Asteroschemasampadae* Parameswaran & Abdul Jaleel, 2012 from the Indian Ocean. Most of the species are pentamerous, but two hexamerous species have been recorded from New Zealand waters (*Asteroschemawrighti* McKnight, 2000 and *Asteroschemabidwillae* McKnight, 2000). Previous studies differentiated *Asteroschema* species based on epidermal ossicle shape and arrangement on the disc and arms, size variance and shape of inner and outer arm spines, and starting point of the second arm spine on the proximal region of the arm (Okanishi and Fujita 2009; Parameswaran and Jaleel 2012). The term epidermal ossicle has been used for small superficial, often granule-like, skeletal elements on the disc and arms (Okanishi and Fujita 2009). Echinoderm skeleton generally develops in the dermis (Byrne 1994), but it is unknown if these ossicles originate in the epidermis or in the dermis, and their possible homology with granules or spines in non-euryalid groups is also unknown. Epidermal thus does not refer to the place of origin of these ossicles, but to their position in adult specimens. *Asteroschema* species have been divided into three groups according to the shape of their epidermal ossicles such as: species with only granular ossicles, with conical and granular ossicles, and domed and with plate-like granular ossicles (Okanishi and Fujita 2009).

The genus *Asterostegus* includes only three species and is morphologically related to the genus *Astroceras* Lyman, 1879, but differs in having the oral shield replaced by several small interradial plates (McKnight 2003; Okanishi and Fujita 2014). Understanding morphological variations and diversity of *Asterostegus* is limited due to a lack of material (Okanishi and Fujita 2014). In the present study, *Asterostegusmaini* McKnight, 2003 from the South China Sea is recorded as the first record since the holotype, but recent studies of *Asterostegus* included detailed descriptions of all three species (Okanishi and Fujita 2014). However, this study includes the intraspecific morphological variation among *A.maini* specimens between the South China Sea and South Pacific waters, and the development of morphological characters relative to size variations. Lastly, the genus *Astrodendrum* is widely distributed from the Indo-Pacific to South Africa, and includes six species. It differs from other genera in the family Gorgonocephalidae by having external ossicles of various shapes on the disc, and by lacking calcareous plates on the lateral disc margin (Okanishi and Fujita 2018).

The present study covers deep waters around the South China Sea (Xisha and Zhongsha Islands) and in the Northwest Pacific region (southwest of Guam Island). Here, we present an account of the *Asteroschema*, *Asterostegus*, and *Astrodendrum* species collection, with descriptions of new species and new records. Our goal is to present a detailed documentation of the morphological features of these species, to complement the limited original descriptions and the lack of figures in the literature. We present the first ever comprehensive tabular key for all species in the genus *Asteroschema*. Two new species are described and seven species are redescribed, including seven new records from the South China Sea and one new record from the Northwest Pacific, all richly illustrated. DNA barcoding was used to identify ophiuroid species in the past two decades (Ward et al. 2008; Hoareau and Boissin 2010; Okanishi et al. 2011, 2018; Okanishi and Fujita 2013). Hence, we use barcoding to test our morphological identifications and to understand the interrelationships within genera. This study also provides biodiversity information of Euryalida species living on seamounts, which may be useful for further studies of euryalid diversity and biogeography.

## ﻿Materials and methods

### ﻿Sample collection

**Figure 1.**
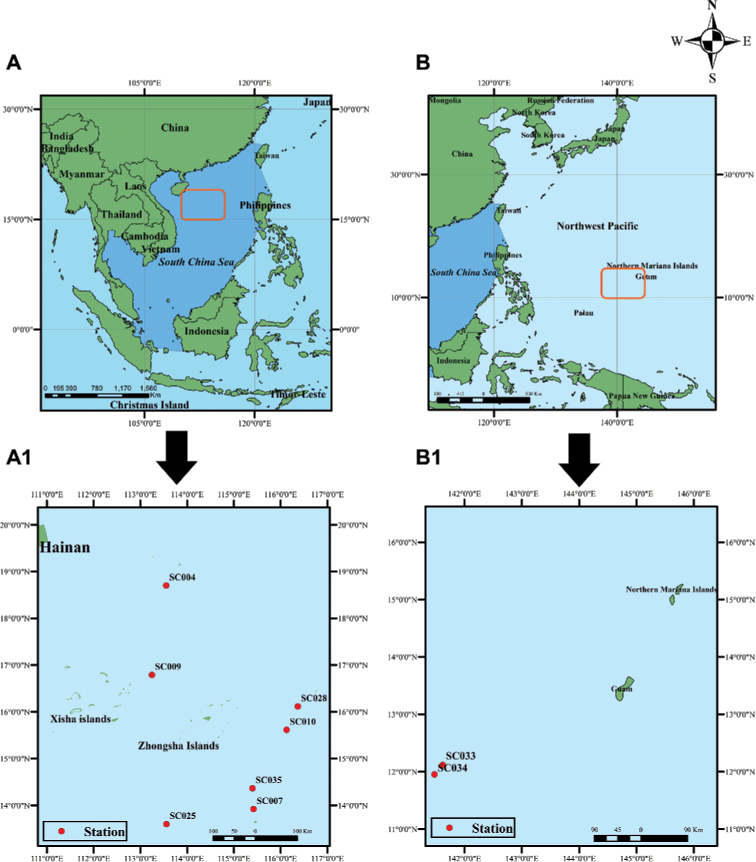
Collecting stations in this study **A, A1** South China Sea (Hainan, Xisha, and Zhongsha Islands) **B, B1** Northwest Pacific (southwest of Guam). Source: International Hydrographic Organization and Sieger (2012).

Ophiuroid specimens were collected by the manned submersible vehicle ‘Shenhaiyongshi’, from 602 to 1920 m depth (Fig. 1). Most of the specimens were frozen at -80°C without preservation fluid, then transported to the Institute of Deep-sea Science and Engineering, Chinese Academy of Sciences (**CAS**), Sanya, China, for further analysis. The samples were sorted and identified by using literature (Pallas 1788; Ljungman 1871; Lyman 1869, 1872, 1875, 1878, 1879, 1882, 1883; Lütken and Mortensen 1889; Alcock 1894; Verrill 1894, 1899; Koehler 1904, 1906, 1907, 1914, 1930; Matsumoto 1911, 1915, 1917; H. L. Clark 1915, 1916b, 1917, 1939, 1941; Mortensen 1924; A. H. Clark 1916a, 1949; Döderlein 1911, 1927, 1930; Murakami 1944; Baker 1980; Guile 1981; Peterson 1985; McKnight 2000; Liao 2004; Mah et al. 2009; Pawson et al. 2009; Parameswaran and Jaleel 2012; Smirnov et al. 2014; Olbers et al. 2015) and by molecular analysis.

### ﻿Morphological analysis

Specimens were photographed through a dissecting stereo microscope (OLYMPUS SZX7) or with a digital camera (Canon EOS 6DII) to identify morphological characters. Arm skeletons were examined with a scanning electron microscope (**SEM**) Phenom ProX. Skeletal elements were prepared by using undiluted NaOCl to dissolve the soft tissue of part of an arm. The excess NaOCl in skeletal elements was removed by repeated flushing with distilled water. Then, the ossicles were mounted on a stub using dissolved carbon tapes. Holotypes, paratypes and all other specimens are deposited at the Institute of Deep-sea Science and Engineering (**CAS**), Sanya, China. The terms used to describe ophiuroids follow previous authors (Martynov 2010; Stöhr 2011, 2012; Okanishi and Fujita 2014, 2018; O’Hara et al. 2017; Hendler 2018; Stöhr and O’Hara 2021). We define granules and spines as articulated ossicles on plates or scales, but tubercles as non-articulated stereom outgrowth, following Stöhr et al. (2012) and Goharimanesh et al. (2021). Following Turner et al. (2021), we utilize the term “pedicellarial bands” for what was previously known as “girdle bands” and for the “girdle hooklets” we use the term “pedicellariae”.

### ﻿Molecular analysis

We extracted DNA from identified specimens by using the TIANamp Marine Animals DNA kit (TianGen, Beijing) following the manufacturer’s protocol. We sequenced cytochrome c oxidase I (COI) and the 16S partial gene for phylogenetic analysis by amplifying primer sets, with suitable PCR cycle (Suppl. material 1: Table S1) (Hoareau and Boissin 2010; Okanishi and Fujita 2013). Total PCR mixture was 50 μL volume, containing 25 μL Premix Taq with 1.25 U Taq, 0.4 mM of each dNTP and 4 mMMg2+ (Ex Taq version, Takara, Dalian, China), 0.5 μM each of the primers and approximately 100 ng template DNA. We performed electrophoresis using a 1.0% agarose gel and the NanoDrop 1000 (Thermo Scientific, Waltham, MA, USA) to assess PCR product quality of the specimens. PCR products were sequenced in both directions on an ABI3730 DNA Analyzer. All new sequences were deposited at NCBI GenBank (Table 1).

**Table 1.** Localities, voucher information, and GenBank accession numbers for all specimens used in this study.

Species	Locality	Voucher number	COI	16S
*Asteroschemashenhaiyongshii* sp. nov.	South China Sea, near Xisha islands	IDSSE-EEB-SW0086	OK044292	OL712208
*Asteroschemabidwillae*	New Zealand	MVF188856	KU895077	-
*Asteroschemarubrum*	South China Sea, near Zhongsha islands	IDSSE-EEB-SW0071	OK044293	OL712209
*Asteroschemarubrum*	South China Sea, near Zhongsha islands	IDSSE-EEB-SW0072	OK044294	OL712210
*Asteroschemarubrum*	South China Sea, near Zhongsha islands	IDSSE-EEB-SW0073	OK044295	OL712211
*Asteroschematubiferum*	New Zealand	MVF188857	KU895076	-
*Asteroschematubiferum*	Mariana Trench, Southeast of Guam Isl.	IDSSE-EEB-SW0078	OK044296	OL712212
*Asteroschematubiferum*	South China Sea, near Zhongsha islands	IDSSE-EEB-SW0106	OK044297	OL712213
*Asteroschematubiferum*	South China Sea, near Zhongsha islands	IDSSE-EEB-SW0077	OK044298	-
Asteroschemacf.lissum	Mariana Trench, Southeast of Guam Isl.	IDSSE-EEB-SW0081	OK044299	OL712207
Asteroschemacf.lissum	South China Sea, near Zhongsha islands	IDSSE-EEB-SW0079	OK044300	-
*Asteroschemasalix*	Australia	TOH_666	HM400451	-
*Asteroschemasalix*	South China Sea, near Zhongsha islands	IDSSE-EEB-SW0082	OK044301	OL712214
*Asteroschema* sp.	South China Sea, near Zhongsha islands	IDSSE-EEB-SW0092	OK044302	OL712215
*Asteroschema* sp.SO2392113	Pacific Ocean: Clarion Clapperton Fracture Zone	SO2392113	MN088049	-
*Asteroschemaclavigerum*	North Atlantic seamounts	-	HM587852	-
*Asteroschemasublaeve*	Canada: British Columbia	RBCM EC00271	HM400328	-
*Asteroschemaajax*	Australia: off Lord Howe Isl.	MVF99759	AB758762	AB605078
*Asteroschemaoligactes*	Off Dominica	MNHN OM62	AB758766	AB758483
*Asteroschemaedmondsoni*	Off Santa Isabel Isl., New Caledonia	MNHN OM13B	AB758831	AB758486
*Asteroschemahorridum*	Off Reunion Isl.	MNHN OM126	AB758764	AB758487
*Asteroschemamigrator*	Off Santa Isabel Isl., New Caledonia	MNHN OM3	AB758765	AB758485
*Ophiocreasambonesicum*	Off Amami-oshima Isl., Kagoshima, Japan	NSMT E-6502	AB758813	AB605084
*Ophiocreasspinulosus*	Caribbean Sea, USA	NMNH OM43	AB758820	AB758490
*Ophiocreasglutinosum*	Off Katsuura, Chiba, Japan	NSMT E-6710	AB758815	AB605086
*Ophiocreasjaponicus*	New Zealand	NIWA T2494	AB758816	AB758488
*Ophiocreassibogae*	South Norfolk Ridge, New Zealand	MV F99763	AB758818	AB605087
*Ophiocreascaudatus*	Sagami Sea, Japan	NSMT E-6259	AB758814	AB605085
*Ophiocreasoedipus*	Off Hachijo-jima Isl., Ogasawara, Japan	NSMT E-6375	AB758817	AB758489
*Asterostegusmaini*	South China Sea, near Xisha islands	IDSSE-EEB-SW0076	OK044303	-
*Asterostegustuberculatus*	Western coast of Madagascar	SMNH-123461	AB758769	AB758515
*Asterostegussabineae*	Madagascar: Off Reunion Island.	SMNH-Type-8333	AB758768	AB758511
*Astrodendrumsagaminum*	Japan: Sagami Sea	NSMT E-5645	AB758795	-
Astrodendrumcf.sagaminum	South China Sea, near Zhongsha islands	IDSSE-EEB-SW0104	OK044304	-
*Gorgonocephaluspustulatum*	New Zealand	MVF188859	KU895114	-
*Gorgonocephalussundanus*	Australia	MVF162682	KU895115	-
*Gorgonocephalusarcticus*	Canada: Nunavut, Barrow Strait	HLC-30309	HM543017	-
*Gorgonocephaluscaputmedusae*	Sweden: Skagerrak	Echin 6305V	MG935270	-
*Gorgonocephaluseucnemis*	Japan: Iwate, Off Miyako	NSMT E-5640	AB758809	-
*Gorgonocephalustuberosus*	Antarctic Sea.	NIWA 38224	AB758811	-
*Gorgonocephaluschilensis*	Antarctic Sea	NIWA 38714	AB758812	-
*Astrogymnotesirimurai*	Seseko Beach, Okinawa, Japan	NSMT E-6716	AB758829	AB605123
*Ophiomyxaanisacantha*	Sagami Sea, Japan	NSMT E-6269	AB758822	AB605124

We constructed two maximum likelihood (**ML**) phylogenetic trees to represent the families Euryalidae and Gorgonocephalidae. Family Euryalidae: to construct the ML tree, we used 12 COI and nine 16S sequences from our collection and additionally 22 COI and 14 16S sequences from GenBank (Table 1). To construct the ML tree for the family Gorgonocephalidae, we used one species from our collection and an additional eight COI sequences from GenBank (Table 1). As outgroup we used COI and 16S sequences of *Astrogymnotesirimurai* Baker et al., 2001 and *Ophiomyxaanisacantha* H. L. Clark, 1911 for the ML trees.

All sequences were aligned using the ClustalW algorithm in MEGA X. When constructing the EuryalidaeML tree, we used the concatenated sequence alignment function in MEGA X to input both COI and 16S sequences. The best-fit substitution model of the COI and 16S gene in the ML trees was the General Time Reversible + Gamma Distributed (GTR + G) model, estimated by the “Find Best DNA/Protein Models” Option of MEGA X. Phylogenetic trees were reconstructed using the maximum likelihood bootstrap method. ML analysis was run with MEGA X, and ML trees were constructed, including 1,000 bootstrap replicates (Kimura 1980; Thompson et al. 1994; Kumar et al. 2016, 2018). The genetic distances were analyzed according to the Kimura 2-parameter model (Kimura 1980), and the standard error of each group was discovered by performing 1,000 bootstrap replications.

### ﻿The following abbreviations are used in the text, tables, and figures

**ap** articular pad of the base;

**ars/ARS** arm spine;

**arsb** arm spine base;

**as** adoral shield;

**asa** arm spine articulation;

**ass/ASS** adoral shield spine;

**au** auricle;

**AUS** Australia;

**CAN** Canada;

**co/CO** conical ossicle;

**COI** Cytochrome C oxide subunit I;

**CS** Caribbean Sea;

**d** dorsal;

**de** depression;

**dist** distal;

**fo** foramina of the base;

**fs** fossa between adjacent tubercles;

**go/GO** granular ossicle;

**goc** granular ossicles coat;

**gs/GS** genital slit;

**hd** head of the apophysis;

**iars** inner arm spine;

**IDSSE** Institute of Deep-sea Science and Engineering;

**irp** interradial plate;

**j** jaw;

**JAP** Japan;

**lac** lateral ambulacral canal;

**MAD** Madagascar;

**lap** lateral arm plate;

**ML** Maximum Likelihood;

**mo** muscle opening;

**mp** median plate;

**msv** manned submersible vehicle;

**NAT** North Atlantic;

**no** nerve opening;

**NWP** North-West Pacific;

**NZ** New Zealand;

**oars** outer arm spine;

**ob** oral bridge;

**os** oral shield;

**PAO** Pacific Ocean;

**pb** podial basin;

**pd** pedicel of the apophysis;

**peb** pedicellarial band;

**po/PO** plate-like ossicle;

**prox** proximal;

**pt** primary tooth of the blade;

**rs/RS** radial shield;

**SCS** South China Sea;

**sh** sheath of the base;

**st** secondary tooth;

**su** sulcus of tubercle head;

**t** teeth;

**TEP** terminal projection;

**tp** tentacle pore;

**v** ventral.

## ﻿Results

**Figure 2.**
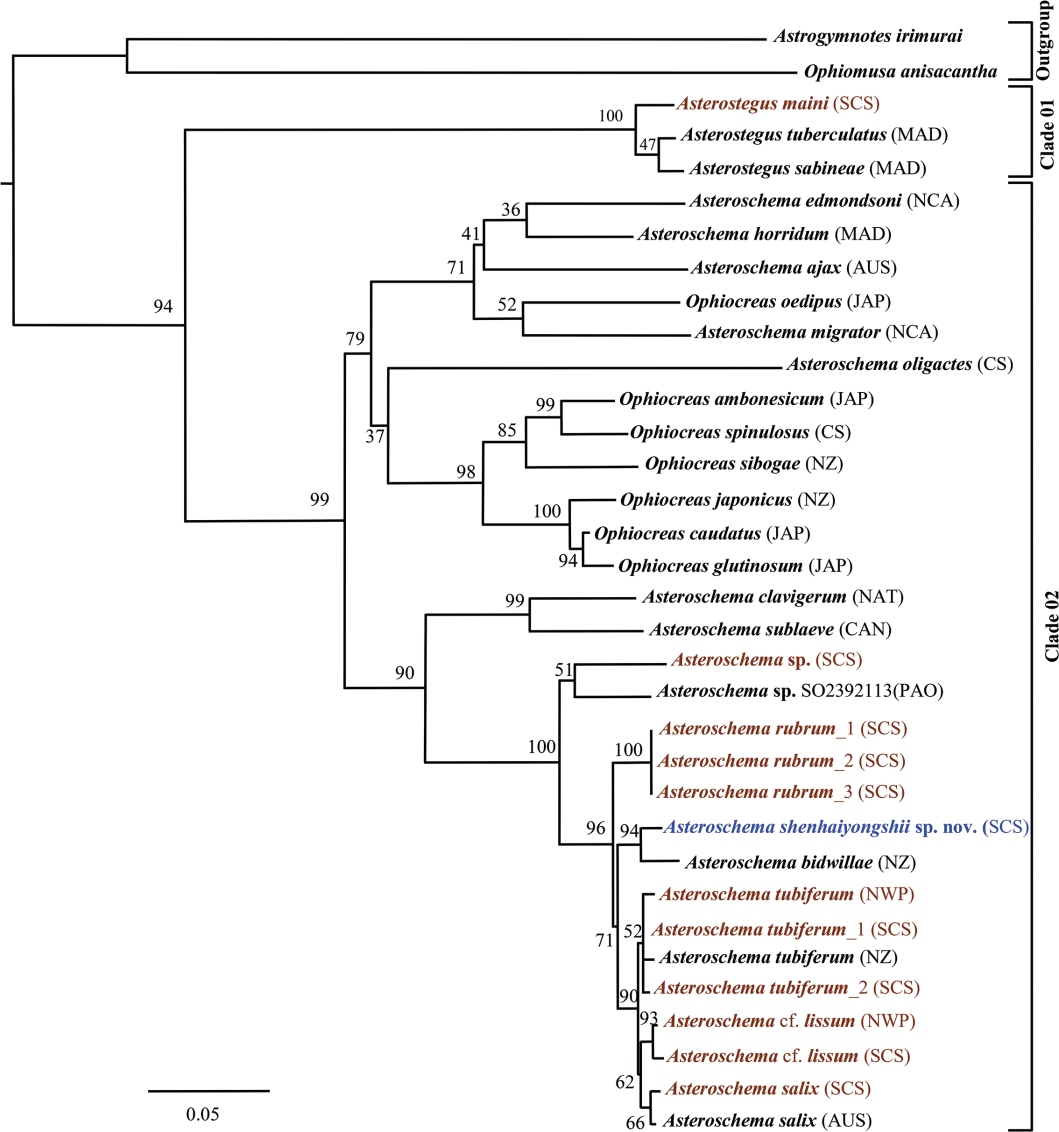
Family Euryalidae, maximum likelihood (ML) tree based on partial COI and 16S sequences (bootstrap support values were generated with rapid bootstrapping algorithm for 1,000 replicates; blue = new species; brown = specimens from this study).

**Figure 3.**
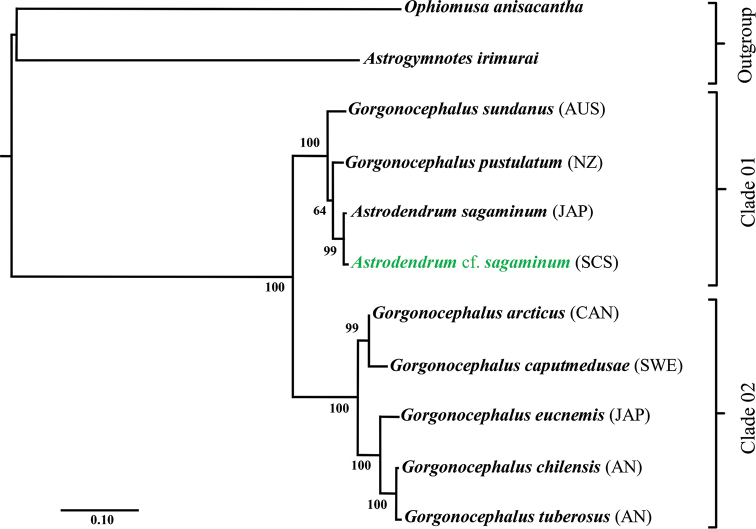
Family Gorgonocephalidae, maximum likelihood (ML) tree based on partial COI sequences (bootstrap support values were generated with rapid bootstrapping algorithm for 1,000 replicates; green = specimen from this study).

Seven species of *Asteroschema* were identified, among them two new to science that are described below. One species of each of the genera *Asterostegus* and *Astrodendrum* were identified, both of them are new to the South China Sea and described below. A tabular key to all species of *Asteroschema* is provided in Table 2. ML phylogenetic trees are presented in Figs 2 and 3, and genetic distances in Suppl. material 2: Tables S2 and Suppl. material 3: Table S3 of most of the species described in the study.

**Table 2.** Tabular key to the species of *Asteroschema* and *Ophiocreas*. Abbreviations: ASS arm segment, ARS arm spine, GO granular ossicles, RS radial shield, CO conical ossicles, PO plate-like ossicles, TP terminal projection, GS genital slits.

Species	Disc diameter and arm length	Epidermal ossicles on the disc		Epidermal ossicles on the arm		ARS length	ARS shape	AS from segment 1^st^ (2^nd^)	Reference
		Dorsal	Ventral	Dorsal	Ventral				
*Asteroschemaajax* A. H. Clark, 1949	13 mm and 300 mm	fine GO; RS narrow, parallel, raised above the disc not meeting at center	fine GO	well-spaced annular bands when dried, covered with GO	covered with GO as dorsal	unknown	unknown	unknown	Clark A. H. (1949)
*Asteroschemaarenosum* Lyman, 1878	8–9 mm and arm length unknown (arm width 4 mm)	coarse GO, 5 grains in 1 mm; RS wide, not meeting at center	coarse GO but near mouth area scattered, jaw covered with GO	coarse GO but denser than on disc	as dorsal	inner ≈ 2 × outer	inner spine: cylindrical, slightly swollen, thorny dark tip	2 (4)	Lyman (1878, H. L. Clark (1941), Pawson et al. (2009)
*Asteroschemabidwillae* McKnight, 2000	5 mm and 50 mm (6 arms)	flat, small, very finely rugose GO, dense at center; RS and interradially (8–10 grains in 1 mm) but slightly spaced beside each RS; more or less extending to center	very small well-spaced GO, except on elongated oral plates	flat, small, very finely rugose GO, slightly spaced toward distal end, almost absent near tip, lateral surface always spaced	GO present on plates near arm base, then naked	inner > outer (inner 1 × ASE, outer 1/3 × ASE length)	inner spine: slightly flattened, denticulate over most of length, and proximal margin beset with small curved spines	2 (8–14)	McKnight (2000), Mah et al. (2009), this study
							outer spine: small and inconspicuous distally both spines flattened and pointed, but hooks absent		
*Asteroschemabrachiatum* Lyman, 1879	6–11 mm and 270 mm (arm base width 3 mm)	dense, uniform GO; RS elevated, extending nearly to center	similar to dorsal, inconspicuous GO, which simulate oral papillae	closely uniformly covered with GO; 6–9 grains in 1 mm	as dorsal	inner > outer (inner spine 2 mm)	inner spine: rough, slightly club-shaped	2 (4)	Lyman (1879, 1882), H. L. Clark (1941)
*Asteroschemaclavigerum* Verrill, 1894	8–12 mm and arm length unknown (arm base width 3–3.5 mm)	small, smooth GO, 6 grains in 1 mm; RS large, extending to center, GO larger than on disc.	smooth skin lacking GO or minute, more spaced GO	small, smooth GO	only base of arm covered with minute, more spaced GO, and rest of arm naked	inner > outer	inner spine: large, long, elevated, and rough with spinules distally; somewhat swollen	3–4 (4–5)	Verrill (1894), Döderlein (1927)
							outer spine: small		
							distally both spines small, slender, acute and nearly equal		
*Asteroschemadomogranulatum* sp. nov.	9 mm and 165 mm	dense, large slightly domed GO, 4 or 5 grains in 1 mm; RS wide, parallel, raised above the disc close together	large polygonal PO except distal half of jaw	large polygonal PO in proximal arm; then slightly separated, decreasing in size GO	arm base concealed by polygonal PO; remainder naked	inner ≈ 2 × outer	inner spine: pointed thorny tip to cylindrical, slightly club shaped, flattened thorny	2 (19–22)	This study
							outer spine: small with thorny tip		
							distally both compound hook with 3–6 secondary teeth		
*Asteroschemaedmondsoni* A. H. Clark, 1949	13 mm and 290 mm	GO & CO; dense GO on proximal half of RS, larger CO with TP on distal half	dense rounded GO	dense PO, swollen in the middle with TP; TP absent in distal end of arm	same as on ventral disc; but smaller	unknown	unknown	unknown	Clark A. H. (1949)
*Asteroschemaelongatum* Koehler, 1914	7–11 mm and 300 mm	strongly excavated, GO rounded, unequal, slightly coarse near disc periphery; RS meeting at center, separated, GO larger and denser than on disc	minute GO, uniform, separated, slightly stronger near periphery	on first few segments GO like on RS then spaced, small, and uniform	GO much smaller and uniform, rapidly becoming fewer and finally disappearing after 30–40 mm from arm base	inner > outer	inner spine: cylindrical, strong, thinner at tip with TP	2 (5)	Koehler (1914), Döderlein (1930)
							outer spine: conical, pointed tip		
*Asteroschemafastosum* Koehler, 1904	6–13 mm and 180–300 mm (arm base width 5 mm)	high, CO including RS	small CO, more rounded, close-set at disc margin	small CO, more close-set than on disc, 6 or 7 grains in 1 mm	flattened CO	inner > outer (inner spine ≤ 4 mm)	inner spine: bluntly conical at arm base, then strongly club-shaped with TP	2 (4–5)	Koehler (1904), Döderlein (1927, 1930), Guile (1981)
							outer spine: cylindrical, thorny tip		
							distally both spines compound hook with 2–4 secondary teeth		
*Asteroschemaflosculus* Alcock, 1894	–	GO & CO; scattered as uniform microscopic GO	uniform microscopic GO	GO & CO; scattered as uniform microscopic GO	uniform microscopic GO	large	unknown	3 (3)	Alcock (1894)
*Asteroschemaglaucum* Matsumoto, 1915	11 mm and 100 mm (4 mm width in arm base)	flat; coarser GO, 6 grains in 1 mm; RS mostly covered except distal end	coarser GO; near apex of jaw less GO	coarser GO, 6 grains in 1 mm, very stout at base, as high as wide	as dorsal	inner > outer	inner spine: cylindrical, club-shaped, rough end	2–3 (8)	Matsumoto (1915)
							distally both spines compound hook with four curved secondary teeth		
*Asteroschemahemigymnum* Matsumoto, 1915	10 mm and 100 mm (3 mm width in arm base)	very fine, smooth, close-set GO	ventral: fine, rather sparse GO in skin, coarse, flat, smooth, pavement-like grains, corresponding to oral papillae	very fine, smooth, close-set GO; 5 grains in 1 mm; GO much finer distalwards and disappear at distal end.	entirely naked; LAP and VAP visible through skin	inner > outer (outer spine ½ × ASE length)	inner spine: cylindrical, club-shaped	2 (5–6)	Matsumoto (1915)
							outer spine: small, cylindrical, enclosed in skin, more or less rough tip		
							distally both spines compound hook with 3–6 secondary teeth		
*Asteroschemahorridum* Lyman, 1879	10–12.5 mm and 160–190 mm	tumid polygonal PO; mostly tall CO with terminal projections	PO & CO higher and thinner at disc periphery	PO, larger on arm base than on disc, 4 PO in 1 mm at arm base; weakly annulated	PO & CO lower and thinner	inner ≈ 2 × outer	inner spine: cylindrical with TP, slightly swollen	1 (1–6)	Lyman (1879, 1882), Baker (1980), Mah et al. (2009), McKnight (2000)
*Asteroschemaigloo* Baker, 1980	5.7 mm and 68.4 mm (1: 12-disc diameter to arm length)	rounded or polygonal domed GO, 4–6 grains in 1 mm length; RS short, obscured, distally visible	closely packed rounded, domed GO	dorsal & ventral: rounded or polygonal domed GO; 4–6 grains in 1 mm length	as dorsal	inner ≈ 2 × outer (inner spine 2/3 × arm width)	inner spine: long, very fine, thorny blunt tip	3: (8–10)	Baker (1980), McKnight (2000)
							distally both spines compound hook with 3–6 secondary teeth		
*Asteroschemainoratum* Koehler, 1906	6–10 mm and 70+ mm	fine, contiguous GO (rounded or slightly conical); RS wider, extending to center	GO density similar to dorsal, slightly developed around GS	fine, rounded or slightly conical, contiguous GO	as dorsal	inner ≈ 1½ × ASE length	inner spine: slightly club-shaped, Swollen toward the end with conical point	2 (5–7)	Koehler (1906), Peterson (1985), Hansson (2001), Smirnov et al. (2014)
							outer spine: smooth, much smaller distally both spines small, but not transforming into a hook		
*Asteroschemaintectum* Lyman, 1878	5–11.5 mm and 280 mm (arm width 3 mm)	fine GO, 6–7 grains in 1 mm; RS long, meeting at center, GO fine than on disc, 8–9 grains in 1 mm	fine GO except jaw	GO scattered, and smaller than on disc.	lateral and ventral side naked or fewer GO	inner > outer	inner spine: blunt, spiniform, not club-shaped	2 (3)	Lyman (1878), H. L. Clark (1941)
*Asteroschemalaeve* (Lyman, 1872)	8.5 mm and 85 mm	flat; fine close-set GO, 7–8 grains in 1 mm; RS mostly covered with GO except distal end	minute, close, smooth GO, fewer GO near apex of jaw	fine close-set GO, 7–8 grains in 1 mm, thin skin, faint brown marking	as dorsal	inner > outer	inner spine: cylindrical, somewhat swollen, rough end	2 (8)	Lyman (1875, 1879), H. L. Clark (1941)
							distally both spines compound hook with 4 curved secondary teeth		
*Asteroschemalissum* H. L. Clark, 1939	7.5 mm and 110 mm	very fine, small GO; 50–60 grains in 1 mm^2^, but noticeably smaller at center and proximal end of RS; RS separated, narrow, straight, parallel, not meeting at center	naked with thin skin	fine GO similar on disc, laterally sparse, but continuous to base of ARS, distally sparse, and naked	naked with thin skin	inner ≈ 2–3 × outer	inner spine: long, thick, thorny tip	2 (9–11)	H. L. Clark (1939) This study
*Asteroschemamigrator* Koehler, 1904	11 mm and 200–300 mm	sparse, domed CO with terminal projections, CO dense on RS & disc margin	few small CO	close-set, small, tumid GO	few GO or CO	inner ≈ 2 × outer	inner spine: cylindrical, swollen, TP outer spine: small, with pointed tip	3 (6–8)	Koehler (1904) Baker (1980) McKnight (2000)
*Asteroschemamonobactrum* H. L. Clark, 1917	8 mm and 80–90 mm (base arm width 2 mm)	GO, flat, slightly raised above arm, near center 7 grains in 1 mm (50 mm^2^), but disc periphery 5–6 grains 1 mm (30 mm^2^)); RS completely covered but rounded ridges appeared when dried	similar GO density as dorsal	GO on arm base similar to disc, then separated, very minute, distally almost naked	GO, from middle slightly naked	inner ≈ 2 × outer	unknown	2 (11–16)	H. L. Clark (1917)
*Asteroschemanuttingii* Verrill, 1899	7 mm and 50 mm	minute rough GO/CO; close-set on RS	few GO near GS; minute rough GO/CO	minute GO/CO, distinct distally	minute rough GO/CO	inner > outer	inner spine: slender, tapering at arm base, then cylindrical, blunt, swollen distally, with TP	1 (1–2)	Verrill (1899)
*Asteroschemaoligactes* (Pallas, 1788)	4–10 mm and 250 mm (length ≈ 17 × disc diameter)	CO	CO	CO; 4–5 CO in 1 mm on ventral arm base	as dorsal	inner > outer	unknown	unknown	Pallas (1788), H. L. Clark (1941)
*Asteroschemarubrum* Lyman, 1879	12 mm and 160 mm	fine, close-set GO, 6–7 grains in 1 mm; RS faintly indicated as flat ridges	fine, close-set GO	fine, close-set GO, 6–7 grains in 1 mm	as dorsal	inner spine maximum length 1.4 mm	inner spine: small spiniform at arm base, then cylindrical, swollen with TP	2 (5–6)	Lyman (1879, 1882), This study
							distally both spines compound hook with secondary teeth		
*Asteroschemasalix* Lyman, 1879	5–8.5 mm and 55–85 mm	flat, fine, close-set GO, 7–8 grains in 1 mm; RS mostly covered with GO except distal end	fine, minute, close-set GO, less GO near apex of jaw	fine, close-set GO, 7–8 grains in 1 mm, thin skin	as dorsal	inner > outer	inner spine: cylindrical, somewhat swollen, rough end	2–3 (11–12)	Lyman (1879), Baker (1980), McKnight (2000), Olbers et al. (2015), This study
							distally both spines compound hook with 4 curved secondary teeth		
*Asteroschemasampadae* Parameswaran & Jaleel, 2012	18 mm and 380–450 mm	spaced CO with terminal projections; RS covered with CO, extending to center	minute, spaced GO	spaced CO with terminal projections, denser at arm base	minute, spaced GO	inner ≈ 2 × outer, inner spine ≤ 2 × ASE (5 mm)	inner spine: bluntly conical at arm base; then cylindrical, with TP at inner edge	2 (4)	Parameswaran and Jaleel (2012)
*Asteroschemashenhaiyongshii* sp. nov.	10 mm and 220 mm (arm base width 3.4 mm)	small, finely rugose, rounded GO, similar in size, 8–9 grains in 1 mm; RS wide, parallel, close together, not meeting at center, distal end of RS raised above disc, swollen at center	GO similar on dorsal, spearhead-shaped teeth. GS narrow concealed with GO	dorsal: dense GO similar to disc; 8–9 grains in 1 mm; distally GO less rounded but dense	less rounded and more polygonal GO, concealing only proximal half of arm, in middle to distal ventral arm surface concealed with widely separated GO decreasing in size to completely naked	inner > outer (inner spine 2.2 mm long)	inner spine: cylindrical, thorny tip to less club-shaped with small sharp thorns on more than half its length	2 (9–11)	This study
							outer spine: small in size with thorny tip similar to inner spine		
							distally both spines similar in size, compound hook with 4 or 5 secondary teeth		
*Asteroschemasubfastostum* Döderlein, 1930	8 mm and 9 × disc diameter	pointed CO, blunt at disc margin, 4–5 grains in 1 mm	smooth hemispherical GO	pointed CO	smooth hemispherical GO	inner > outer	unknown	unknown	Döderlein (1930)
*Asteroschemasublaeve* Lütken & Mortensen, 1889	12 mm and 300 mm (arm base width 5 mm)	round, rugose GO variable size; RS covered with larger GO than on the disc	smooth, small GO, few GO lateral at jaw	round, rugose GO variable size, smaller at lateral side, larger on dorsal surface	naked	inner ≈ 2 × outer; in middle (inner ≈ 4 × outer)	inner spine: elongated, club shaped, enclosed with thick skin	2–3 (3–4)	Lütken and Mortensen (1889)
*Asteroschemasulcatum* Ljungman, 1872	5 mm and arm length unknown	dense, small GO (9–15 grains in 1 mm); RS narrow, not meeting at center	dense, small GO, teeth rounded or distally lobed	dense, small GO	as dorsal	inner > outer	inner spine: strongly thorny tip, swollen, bent club shaped	(3–12)	Ljungman (1872), Lyman (1875)
*Asteroschematenue* Lyman, 1875	6 mm and 200 mm (arm base width 1.5 mm)	closely, smooth GO (8–9 grains in 1 mm); RS narrow, meeting at center and GO little coarser	coarser GO; large GO in jaw	slender arms; similar to dorsal disc, distally GO much finer and more scattered	as dorsal	inner > outer, inner spine 1 mm long in middle half	inner spine: spiniform at arm base, then large, fine thorny, club-shaped	1 (3)	Lyman (1875), H. L. Clark (1915)
*Asteroschematubiferum* Matsumoto, 1911	14–16 mm and 230–300 mm	closely and evenly, small rounded or polygonal GO; RS narrow, not meeting at center but convergent	entirely covered with dense GO, slightly large, rounded GO corresponding to oral papillae	GO similar to disc, 4–5 grains in 1 mm	distally GO smaller, widely spaced on ventral side	inner > outer	inner spine: cylindrical, initially tapering to a blunt, thorny tip, middle club-shaped with small sharp thorns, first 10–12 covered by sheath	2 (7)	Matsumoto (1911, 1915), Baker (1980), McKnight (2000), This study
							outer spine: small, pointed tip		
							distally both spines compound hook with secondary teeth		
*Asteroschematumidum* Lyman, 1879	8–13 mm and 120–180 mm	dense, rounded GO proximally, CO at disc margin regularly spaced pointed; RS covered with CO, extending to center	similar to dorsal but lower	regularly spaced pointed, CO; 4 grains in 1 mm, rarely touching each other	as dorsal	inner > outer, inner spine ≤2 mm long	inner spine: rough, slightly club-shaped	2 (3)	Lyman (1879, 1882) Koehler (1904)
*Asteroschemavicinum* Koehler, 1907	7 mm and 93 mm	fine GO	fine GO, more than 9 grains in 1 mm, GO much larger around jaw	fine GO	as dorsal	½ × ASE length	both sub-equal, fairly short, same morphology along the arm	- (2)	Koehler (1907)
*Asteroschemawrighti* McKnight, 2000	6.5 mm and at least 6 × disc diameter	fine and uniform GO, 8–10 grains in 1 mm; RS elongated, meeting at center	thin smooth skin, occasional small GO; relatively large irregular PO in GS	fine and uniform GO; 8–0 grains in 1 mm; extending to lateral surface	occasional small GO, thin and smooth skin	inner > outer, inner arm spine up to 2 × ASE length	inner spine: long, finely thorny, slightly club-shaped	2(5–6)	McKnight (2000)
							outer spine: relatively small and smooth		
							distally both spines compound hook with secondary teeth		
*Asteroschemayaeyamense* Murakami, 1944	7 mm and 150 mm	dense, coarse CO	rounded GO	regularly spaced CO, distally swollen GO	as dorsal	inner > outer	inner spine: long, cylindrical with TP	2 (3–6)	Murakami (1944), Liao (2004)
*Ophiocreasambonesicum* Döderlein, 1927	27–30 mm and 350–390 mm	coarse, thick, naked skin (when dry widely separated GO visible); RS narrow, not meeting at center	as dorsal	naked skin, when dry widely separated GO visible), annular band	naked skin, when dry widely separated GO visible)	inner ≈ 3 × outer; in middle 2 × ASS length	inner spine: elongated, thick, enclosed with thick skin, cylindrical, club-shaped	2 (8–12)	Döderlein (1927)
							outer spine: cylindrical, pointed tip		
							distally both spines compound hook with 2–3 secondary teeth		
*Ophiocreascarnosus* Lyman, 1879	15 mm and 200 mm (arm base width 7 mm)	thick, soft wrinkled skin; RS rounded distal end, narrow, meeting at disc center	as dorsal	smooth, soft wrinkled skin	as dorsal	inner ≈ outer, inner ≈ 3 mm long	inner spine: short, enclosed by thick skin, cylindrical, thorny tip	2 (6)	Lyman (1879)
							outer spine: cylindrical, thorny tip		
*Ophiocreascaudatus* Lyman, 1879	22–25 mm and 300–420 mm (arm base width 5.5–7.5 mm)	covered with thick skin, when dry micro-GO visible; RS narrow, raised above the disc, meeting at center	as dorsal	covered by thick skin, when dry micro-GO visible at arm base, annular band.	as dorsal	inner 3 mm longer in middle	inner spine: elongated, enclosed by skin, stout, thorny tip	2 (10–13)	Lyman (1879, 1882), Matsumoto (1917), Döderlein (1911), H. L. Clark (1949)
							outer spine: short, peg-like		
*Ophiocreasoedipus* Lyman, 1879	5–12 mm and 70–250 mm (arm base width 3.5 mm)	thin skin with small, fine GO; RS narrow, closer together, extending to disc center	small, closely set, rounded GO or naked, GS wide	thin skin with fine GO, first 5–8 ASS swollen	as dorsal	inner 1 × ASS length, outer ½ × ASS length	inner spine: slender, elongated, enclosed by skin, blunt, thorny tip	2 (6–9)	Koehler (1904, 1909), Lyman (1879, 1882), H. L. Clark (1915), Baker (1980), Peterson (1985), McKnight (2000)
							outer spine: short, cylindrical, pointed		
							distally both spines compound hook with 6 secondary teeth		
*Ophiocreasgilolense* (Döderlein, 1927)	22 mm and 290 mm (arm base width 9 mm)	naked skin; RS meeting at center	as dorsal	naked skin, annular band	as dorsal	inner ≈ 2 × outer; in middle 2 × ASS length	inner spine: elongated, slender, cylindrical	2 (3–4)	Döderlein (1927)
							outer spine: cylindrical, pointed tip		
*Ophiocreasglutinosum* (Döderlein, 1911)	17 mm and unknown (arm base height 9 mm)	dense small GO (10 grains in 1 mm); RS large (nearly covering whole disc), long, close to each other, meeting at center	as dorsal	thick arms, thick skin covers arm plates completely, proximally dense small GO (10 grains in 1 mm), distally separated,	as dorsal	inner 1½ × ASS length, outer ≈ 1/3 × inner	inner spine: slender, elongated, swollen thorny tip	2 (6–7)	Döderlein (1911)
							outer spine: small		
							distally both spines compound hook with 2–3 secondary teeth		
*Ophiocreasjaponicus* Koehler, 1907	4–33 and 210–655 mm (arm base width 7 mm)	smooth, thin, small specimen with dense GO coverage (4 mm disc diameter); RS thick, raised above the disc, meeting at disc center	as dorsal	smooth, thin, naked skin, annular, (small specimen with dense GO coverage (4 mm disc diameter)	somewhat fewer scattered GO	inner ≤6 mm, 3 × ASS length; outer 1 × ASS length	inner spine: elongated, thick base, cylindrical, thorny tip	2 (3–12)	Koehler (1907), Döderlein (1911), Matsumoto (1917), McKnight (2000)
							outer spine: cylindrical, pointed tip		
							distally both spines compound hook with 2–3 secondary teeth		
*Ophiocreaslumbricus* Lyman, 1869	4.5–17 mm and 50–240 mm (arm base width 2.5 mm)	covered by separated micro-thorny GO; RS meeting at center	as dorsal	covered by separated micro-thorny GO annular band	as dorsal	inner ≤ 1½–2 × ASS length outer 1 × ASS length	inner spine: cylindrical, enclosed by skin, blunt, rough surface, thorny tip	2 (4)	Lyman (1869)
							outer spine: cylindrical, enclosed with skin, blunt, rough surface, thorny tip		
*Ophiocreasmindorense* (Döderlein, 1927)	12–23 mm and 160–480 mm (arm base width 3–5.5 mm)	smooth, dense GO (8 grains in 1 mm); RS narrow, closer together, extending to disc center	as dorsal	similar to disc, smooth, dense GO (8 grains in 1 mm); GO absent on ventral side of younger specimens	as dorsal	inner 3–5.5 mm, in middle 2½ –3 × ASS length; outer 1 × ASS length	inner spine: slender, elongated, swollen thorny tip	2 (8–11)	Döderlein (1927)
							outer spine: thick arm base with pointed tip		
*Ophiocreasmortenseni* Koehler, 1930	7.5–25 mm and 110–400+ mm (arm base height 6 mm)	covered by thick, wrinkled, or folded skin; RS narrow, extending to disc center	covered by plate-like ossicles	skin thicker than dorsal, mostly thickened near arm spines, arched	as dorsal	inner 1 × ASS length; outer 2/3 × inner	inner spine: slightly flattened, rough in upper half, thorny tip	2 (4–7)	Koehler (1930), McKnight (2000)
							outer spine: short, cylindrical, thorny pointed		
							distally both spines compound hook with 3 or 4 secondary teeth		
*Ophiocreassibogae* Koehler, 1904	14–28 mm and 300–350 mm (disc diameter × 30) (arm base width 5 mm)	naked skin; RS narrow, parallel, not meeting at center	as dorsal	naked skin, annular band	as dorsal	inner ≈ 2 × outer, in middle 2 × ASS length	inner spine: elongated, slender, cylindrical, club-shaped, finely rugose	2 (3–11)	Koehler 1904, H.L. Clark 1916b, Mortensen 1924, Döderlein 1927, Baker 1980, McKnight (2000)
							outer spine: cylindrical, pointed tip		
							distally both spines compound hook with 2–4 secondary teeth		
*Ophiocreasspinulosus* Lyman, 1883	8–17 mm and 60–550 mm (arm base width 3 mm)	naked skin; RS strongly marked ridges with short, stout blunt spines, meeting at center	as dorsal	higher than wide, naked skin, annular band blunt spine at each pair of ASS	as dorsal	inner ≈ 2 mm, equal in size in proximal arms	inner spine: short, blunt, rough surface, cylindrical	2 (3–4)	Lyman (1883), H. L. Clark (1915, 1941)
							outer spine: cylindrical, pointed tip, distally slender		
							distally both spines compound hook with 2 secondary teeth		
*Ophiocreaswillsi* McKnight, 2000	16 mm and 380 mm	covered by thick, wrinkled, or folded skin; RS narrow, extending to disc center, dense small GO cover in the center	covered by well separated small GO.	granulation similar to the disc, extending to the lateral arm, GO coverage dense proximally, but scattered distally	first 3–5 ASS with few GO, then naked	inner 1½ × ASS, outer shorter than ASS	inner spine: slightly flattened, enclosed by skin, rough, blunt tip	2 (2–3)	McKnight (2000)
							outer spine: short, cylindrical, thorny pointed		
							distally both spines compound hook with 3 or 4 secondary teeth		

### ﻿Molecular phylogenetic analysis

In total, 34 COI sequences trimmed to 592 bp and 25 16S sequences trimmed to 453 bp were obtained after removing ambiguous aligned sites and successfully reconstructing an ML tree for the studied Euryalidae (Fig. 2). Two main clades were detected within the ML tree of Euryalidae (clade 01: genus *Asterostegus*; clade 02: genera *Asteroschema* and *Ophiocreas*). Overall average genetic distances of COI between two clades were 10.04±1.34% SE (*Asteroschema* and *Ophiocreas*) and 3.08±0.75% SE (*Asterostegus*). The maximum value between two clades was 24.79%. Species from the genera *Asteroschema* and *Ophiocreas* separated into two subclades within main clade 02, but *Asteroschemaoligactes* (Pallas, 1788), *A.migrator* Koehler, 1904, *A.edmondsoni* A. H. Clark, 1949, *A.ajax* A. H. Clark, 1949 and *A.horridum* Lyman, 1879 clustered with *Ophiocreas* species. Genetic distance between *Asteroschemabidwillae* and *Asteroschemashenhaiyongshii* sp. nov. was 2.59±0.67% SE (Suppl. material 2: Table S2).

A total of 11 COI sequences trimmed to 730 bp were obtained after removing ambiguous aligned sites, and successfully reconstructing an ML tree for the genera *Gorgonocephalus* and *Astrodendrum* (Fig. 3). Two clades were detected between the species. Clade 1 consists of *Astrodendrumsagaminum* (Döderlein, 1902), *Gorgonocephaluspustulatum* (H. L. Clark, 1916), and *G.sundanus* (Döderlein, 1927). Clade 2 consists of *Gorgonocephalusarcticus* Leach, 1819, *G.eucnemis* (Müller & Troschel, 1842), *G.chilensis* (Philippi, 1858), and *G.tuberosus* Döderlein, 1902. Overall average genetic distances of COI between two clades were 2.88±0.58% SE (clade 01) and 5.39±0.87% SE (clade 02). The maximum value between the two clades was 15.21%. Genetic distance between *Astrodendrumsagaminum* (AB758795) and Astrodendrumcf.sagaminum (OK044304) was 0.69±0.30% SE (Suppl. material 3: Table S3).

### ﻿Taxonomic account

#### Class Ophiuroidea Gray, 1840

**Superorder Euryophiurida O’Hara, Hugall, Thuy, Stöhr & Martynov, 2017**

**Order Euryalida Lamarck, 1816**

**Family Euryalidae Gray, 1840**

##### Genus *Asteroschema* Örsted & Lütken in Lütken, 1856

###### ﻿ Asteroschema domogranulatum sp. nov.

Taxon classificationAnimaliaEuryalidaEuryalidae

4B9157AD-F3EA-59EB-8446-6499E1679AE6

http://zoobank.org/68786758-AC50-415B-8835-1CCE871304E5

Figures 4 , 5

####### Material examined.

***Holotype***: China • 1 specimen; South China Sea, East of Zhongsha Islands, seamount; 16°22.11'N, 113°6.01'E; depth 1742 m; 09 Aug. 2020; Collecting event: stn. SC028; ‘Shenhaiyongshi’ msv leg; preserved in -80 °C; IDSSE-EEB-SW0089.

***Paratypes***: China • 2 specimens; same data as for holotype; IDSSE-EEB-SW0090, IDSSE-EEB-SW0091.

####### Diagnosis.

**Figure 4.**
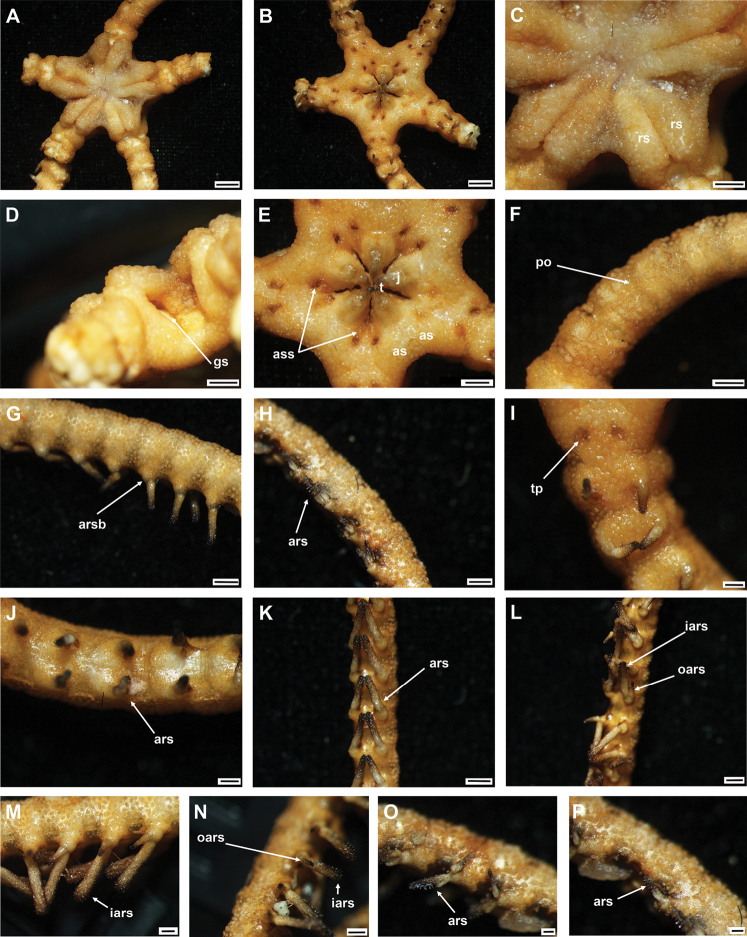
*Asteroschemadomogranulatum* sp. nov., holotype (IDSSE-EEB-SW0089) **A** dorsal view **B** ventral view **C** dorsal disc **D** lateral disc **E** ventral disc **F** dorsal arm (proximal) **G** lateral arm (proximal) **H** lateral arm (distal) **I** ventral arm (base) **J, K** ventral arm (proximal) **L** ventral arm (middle) **M, N** arm spines (middle) **O, P** arm spines (distal). Abbreviations: **ars** arm spine, **arsb** arm spine base, **as** adoral shield, **ass** adoral shield spine, **gs** genital slit, **iars** inner arm spine, **j** jaw, **oars** outer arm spine, **po** plate-like ossicle, **rs** radial shields, **t** teeth, **tp** tentacle pore. Scale bars: 2 mm (**A, B**); 1 mm (**C–G, K, L**); 500 µm (**H–J, M, N**); 200 µm (**O, P**).

Radial shields straight, parallel, close together, and raised above the disc and arms (Fig. 4A). Disc concealed by large polygonal, slightly domed granular ossicles (Fig. 4C). Jaws elongated, apex covered with few granular ossicles, but distal half naked. Ventral disc covered with large polygonal plate-like ossicles but naked around distal half of jaws (Fig. 4E). Dorsal and lateral surface of arms covered with plate-like or granular ossicles but dense only on few arms segments beyond the arm base (Fig. 4F–H). Ventral surface of the arm naked except arm base (Fig. 4I–L).

####### Description of holotype.

Disc diameter 9 mm, length of arms 165 mm, arm base width 2.8–3.0 mm (Fig. 4).

***Disc*.** Disc star-shaped, pentagonal, raised high above the arms, incised interradially and swollen on radial shields (Fig. 4A, B). Disc concealed by dense, large, polygonal, slightly domed ossicles (three or four grains in 1 mm; Fig. 4C). Radial shields bar-like, long, parallel, straight, adjacent pairs separated by narrow interradial disc, raised above the disc, and almost extending to center (Fig. 4C). Domed ossicles on distal half of radial shields larger (two or three grains in 1 mm) than in center (four grains in 1 mm; Fig. 4C). Genital slits narrow, vertical on interradii, dorsal half covered with ossicles similar to dorsal disc, ventral half similar to ventral disc (Fig. 4D). Jaws elongated, apex covered with few granular ossicles, but distal half naked (Fig. 4E). At apex of jaw a bluntly pointed tooth, at lateral edges a few granules that resemble lateral oral papillae (Fig. 4E). Ventral disc covered with large polygonal plate-like ossicles (three or four grains in 1 mm) except distal half of jaws (Fig. 4E). Adoral shields large but completely concealed by ossicles. Oral shields not discernible, and naked adoral shield spine (Fig. 4B, E).

***Arms*.** Arms slender, arched at base, sub-cylindrical, increasingly cylindrical and narrower distalwards (Fig. 4F–H). Dorsal surface of arm base covered with large polygonal plate-like ossicles (three or four grains in 1 mm), then decreasing in size (five or six grains in 1 mm) and separated along the arm (Fig. 4F–H). Lateral plate covered with granular or plate-like ossicles, larger than on dorsal surface, and continuing to near base of arm spine (Fig. 4G, H). Distal half of arm laterally and dorsally covered with similar in size, separated granular ossicles (seven or eight grains in 1 mm; Fig. 4H). Ventral surface of arm base covered with polygonal plate-like ossicles (five or six grains in 1 mm), but after few arm segments from arm base completely naked (Fig. 4I–L). Tentacle pore at first arm segment without arm spine, but with small extended tube or sheath (Fig. 4I). Single arm spine from second arm segment with a second arm spine from nineteenth or twenty-second arm segment (Fig. 4I–L). Inner arm spine initially tapering to pointed thorny tip, middle half cylindrical, slightly club-shaped, one and a half arm segment in length, flattened, thorny (Fig. 4K–N). Outer arm spine half as long as inner spine in middle region, with thorny tip (Fig. 4N). Both arm spines similar in size at distal end, a compound hook with 3–6 secondary teeth (Fig. 4O, P).

***Color*.** In live specimen, light brown color (Fig. 4).

####### Ossicle morphology of one paratype.

**Figure 5.**
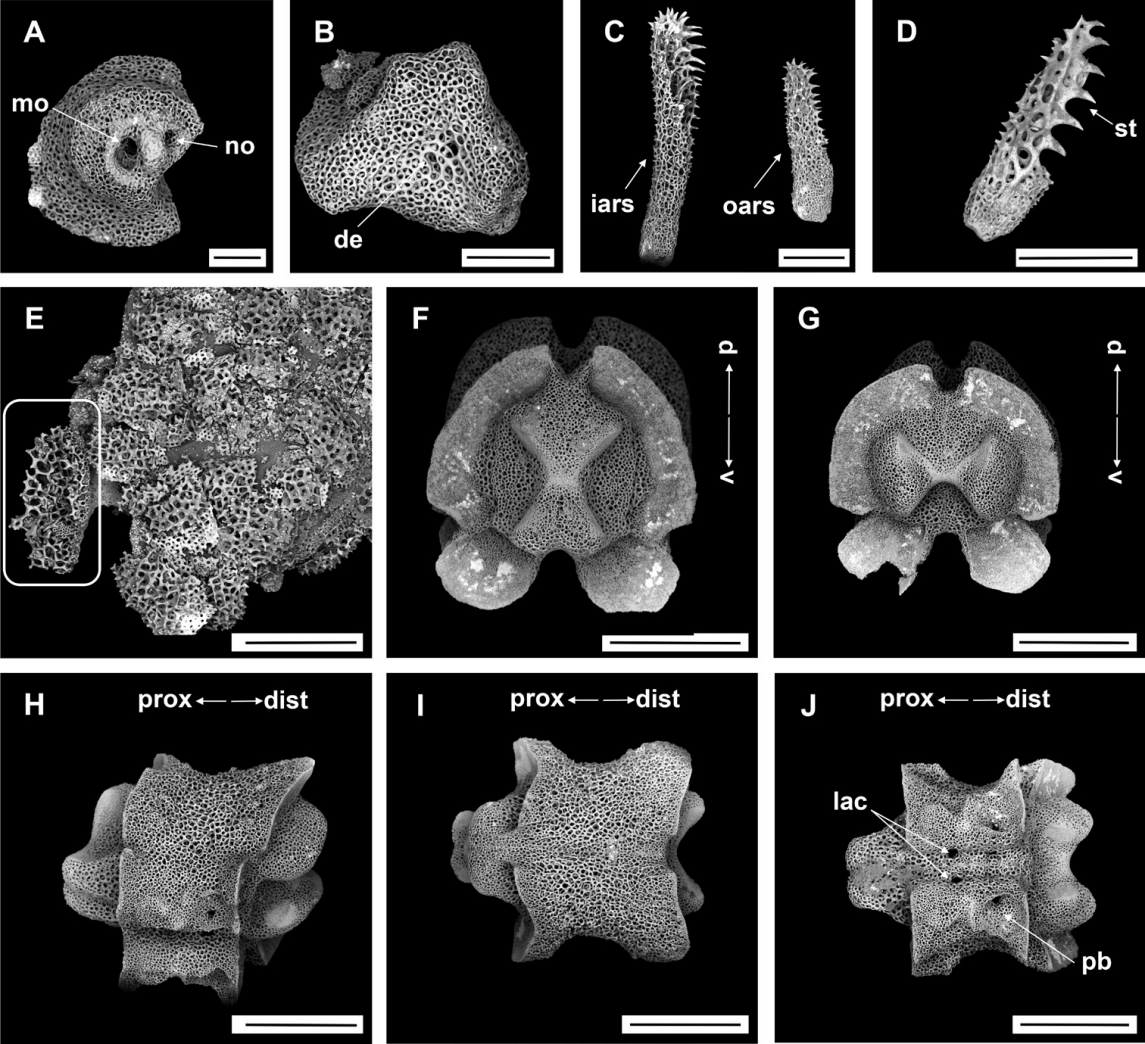
*Asteroschemadomogranulatum* sp. nov., paratype (IDSSE-EEB-SW0090) **A, B** lateral arm plate (external, internal) **C** arm spines (middle) **D** arm spine (distal) **E** skin from dorsal arm base, insert frame shows polygonal plate-like large ossicle **F–J** vertebrae **F** proximal view **G** distal view **H** lateral view **I** dorsal view **J** ventral view. Abbreviations: **d** dorsal, **de** depression, **dist** distal, **iars** inner arm spine, **lac** lateral ambulacral canals, **mo** muscle opening, **no** nerve opening, **oars** outer arm spine, **pb** podial basin, **prox** proximal, **st** secondary teeth, **v** ventral. Scale bars: 800 µm (**F–J**); 500 µm (**C**); 300 µm (**B, D, E**); 200 µm (**A**).

IDSSE-EEB-SW090: Lateral arm plate curved around vertebrae, with strong curved rib with one arm spine articular structure, with single, completely separated large muscle and nerve openings (Fig. 5A). A depression on inner side of lateral arm plate (Fig. 5B). In proximal and middle half of arm inner arm spine slightly swollen, flat, and thorny on distal arm. Outer arm spine nearly half the size of the inner one with thorny tip (Fig. 5C). Distally, both spines changing into compound hook with secondary teeth (Fig. 5D). Arm concealed by polygonal large granular or plate-like ossicles (Fig. 5E). Vertebrae with streptospondylous articulation, with deep groove between proximal and distal end, dorsally a median longitudinal furrow, ventrally with deep median longitudinal groove containing lateral ambulacral canals, no oral bridge (Fig. 5F–J).

####### Paratypes variations.

Disc diameter 6.5 and 8 mm, and both basically identical to holotype. However, the segment at which the second arm spine first appeared varied (14–20 free segments), but is considered intraspecific variation.

####### Distribution and habitat.

1742 m depth. Zhongsha Islands, the South China Sea. Attached to coral host.

####### Etymology.

The species name is derived from the Latin words *domus*, meaning dome, and *granulatus*, meaning granulated, referring to the domed granular ossicles on the disc.

####### Remarks.

The here examined new species was collected on a deep-sea seamount, attached to an unidentified coral species. It concurs with the group that has domed and plate-like granular ossicles, in the genus *Asteroschema*. This clade included only one species, prior to this study (*Asteroschemaigloo* Baker, 1980). Large polygonal plate-like ossicles were the most significant morphological character for delimiting most of the other *Asteroschema* species from *A.domogranulatum* sp. nov. (Table 2).

*Asteroschemadomogranulatum* sp. nov. strongly resembles *A.igloo*. They are similar in size according to McKnight’s (2000) description (8 mm disc diameter). Therefore, here we include a comprehensive morphological analysis to distinguish *A.domogranulatum* sp. nov. from *A.igloo* such as (see also Table 2): in *A.domogranulatum* sp. nov. radial shields raised above the arms and disc, straight, parallel, with narrow gap, whereas in *A.igloo* distal ends of radial shields much wider apart, converging to center, in *A.domogranulatum* sp. nov. polygonal granular ossicles on dorsal disc, in center smaller than at distal edge, but in *A.igloo* concealed by polygonal or rounded domed ossicles, and in center large, domed, rounded ossicles, in *A.domogranulatum* sp. nov. teeth pointed but in *A.igloo* ventralmost one pointed and others blunt spearhead-shaped, in *A.domogranulatum* sp. nov. ventral disc covered with polygonal plate-like ossicles, and distal half of jaw naked but in *A.igloo* completely covered with compact polygonal or rounded domed ossicles, in *A.domogranulatum* sp. nov. only dorsal and lateral surface covered with plate-like or granular ossicles, dense only on few arm segments from arm base, and naked ventral arm except arm base but in *A.igloo* whole arm covered with dense, rounded or polygonal domed ossicles, in *A.domogranulatum* sp. nov. inner arm spine slightly swollen, blunt, flattened, and outer arm spine with thorny pointed tip but in *A.igloo* inner arm spine swollen, blunt, and outer arm spine with smooth pointed tip, in *A.domogranulatum* sp. nov. start of first arm spine at second arm segment, and second arm spine at nineteenth or twenty-second arm segment but in *A.igloo* first arm spine from third arm segment, and second arm spine starts at eighth or tenth arm segment (McKnight 2000). The most significant morphological characters of *A.domogranulatum* sp. nov. were the appearance of the radial shields, and the granulation of ventral disc and arms (Fig. 4).

###### ﻿ Asteroschema shenhaiyongshii sp. nov.

Taxon classificationAnimaliaEuryalidaEuryalidae

95009617-0B1B-5144-98DC-CF83315443E6

http://zoobank.org/A5459AA8-D154-47F1-833F-710652EF9636

Figures 6 , 7

####### Material examined.

***Holotype***: China • 1 specimen; South China Sea, Northeast of Xisha Islands archipelago; 18°41.95'N, 113°33.08'E; depth 1070 m; 29 Mar. 2018; Collecting event: stn. SC004; ’Shenhaiyongshi’ msv leg; preserved in -80 °C; GenBank: OK044292, OL712208; IDSSE-EEB-SW0086.

***Paratype***: China • 1 specimen; South China Sea, Southeast of Zhongsha Islands; 13°55.30'N, 115°25.44'E; depth 1111 m; 04 Aug. 2020; Collecting event: stn. SC007; ‘Shenhaiyongshi’ msv leg; preserved in -80 °C; IDSSE-EEB-SW0087.

####### Diagnosis.

**Figure 6.**
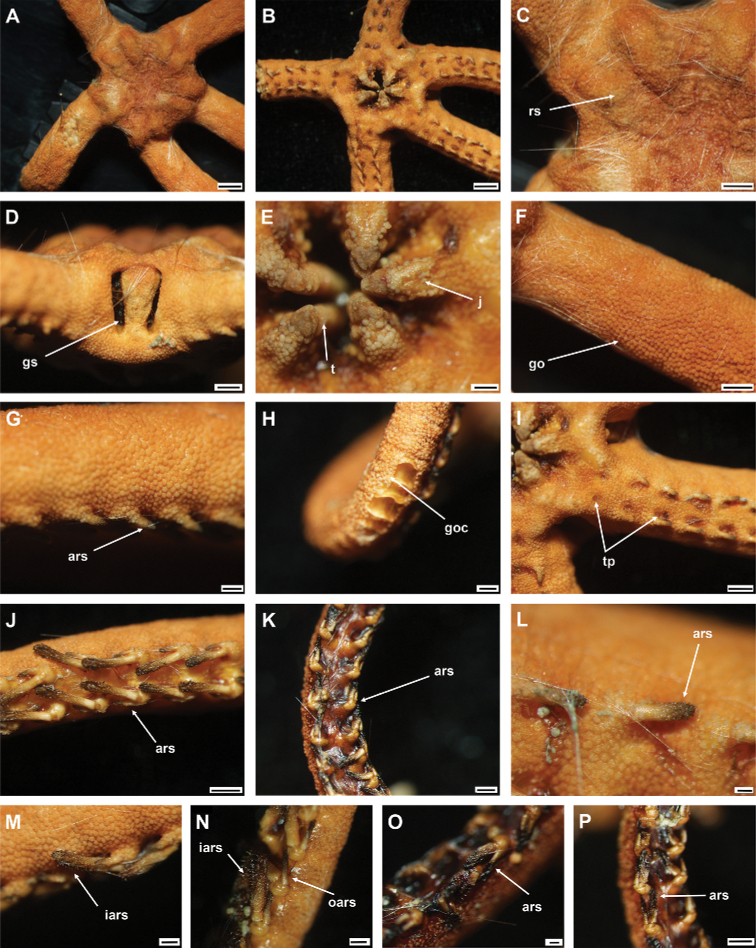
*Asteroschemashenhaiyongshii* sp. nov., holotype (IDSSE-EEB-SW0086) **A** dorsal view **B** ventral view **C** dorsal disc **D** lateral disc **E** oral frame **F** dorsal arm (base) **G** lateral arm (proximal) **H** dorsal arm (distal) **I** ventral arm (base) **J** ventral arm (middle) **K** ventral arm (distal) **L** arm spine (arm base) **M, N** arm spines (middle) **O, P** arm spines (distal). Abbreviations: **ars** arm spine, **go** granular ossicle, **goc** granular ossicles coat, **gs** genital slit, **iars** inner arm spine, **j** jaw, **oars** outer arm spine, **rs** radial shield, **t** teeth, **tp** tentacle pore. Scale bars: 2 mm (**A, B**); 1 mm (**C, D, F, I, J**); 500 µm (**E, G, H, K, M, N, P**); 200 µm (**L, O**).

Disc raised high above the arm, concealed by highly dense, small, rounded, finely rugose granular ossicles (Fig. 6A–C). Jaws narrow, elongated, concealed by slightly larger, less rounded granular ossicles (Fig. 6E). Arm surface concealed by granular ossicles similar to disc, but ventral surface of arm concealed by less rounded, more polygonal granular ossicles (Fig. 6F–H). Inner arm spine cylindrical, slightly club-shaped, with small sharp thorns on more than half its length (Fig. 6L–N).

####### Description of holotype.

Disc diameter 10 mm, length of arms 220 mm, arm base width 3.3–3.5 mm (Fig. 6).

***Disc*.** Disc more or less pentagonal, raised high above arm, and swollen in center (Fig. 6A). Entire disc concealed by highly dense, small, rounded, finely rugose granular ossicles (eight or nine grains in 1 mm; Fig. 6A–C). Granular ossicles similar in size and shape from center to periphery of disc (Fig. 6C). Radial shields wide, straight, close together, but not meeting in center, and completely concealed by dense granulation (Fig. 6C). Genital slits narrow, vertical at interradii and densely covered with less rounded granular ossicles (Fig. 6D). Jaws narrow, elongated, concealed by slightly larger less rounded granular ossicles (Fig. 6E). At apex of jaw a blunt, spearhead-shaped tooth, and granular ossicles all over oral plates (Fig. 6E). Ventral disc densely covered with granular ossicles similar to dorsal disc (seven or nine grains in 1 mm) but less rounded around distal end of jaw and adoral shields (Fig. 6E). Adoral shields large, with curved edge, and connected to first arm segment. Oral shields not discernible and adoral shield spine covered by granular ossicles (Fig. 6E).

***Arms*.** Arm width comparatively large in relation to body size, not arched, sub-cylindrical, width unchanged from base to middle half of arm (Fig. 6A, F). From middle to distal end, arm tapering slightly and more cylindrical (Fig. 6F–H). Dorsal and lateral arm surface concealed by dense, finely rugose, rounded granular ossicles similar to disc (eight or nine grains in 1 mm), continuing to distal end of arm (Fig. 6F–H). Distal half of dorsal and lateral arm concealed by less rounded, dense granular ossicles (eight or nine grains in 1 mm; Fig. 6H). Lateral arm plates on proximal to middle half of arm concealed by granular ossicles, including on base of arm spine, but on distal end only lateral arm plates concealed (Fig. 6G–K). Ventral surface of arm concealed by dense granular ossicles, similar to ventral disc, less rounded and more polygonal, but only covering proximal half of arm (seven or nine grains in 1 mm; Fig. 6I). Middle to distal end of ventral arm surface concealed by widely separated, in size decreasing granular ossicles (three or four grains in 1 mm) to completely naked (Fig. 6J, K). First free tentacle pore without arm spine (Fig. 6I). First arm spine appears at second arm segment, with short blunt tentacle scale. Second arm spine appears at nineth or eleventh segment (Fig. 6I, J). Inner arm spine initially tapered to pointed thorny tip, one arm segment in length, middle half cylindrical, less club-shaped, with small sharp thorns on more than half of spine length (Fig. 6L–N). Outer spine smaller, with thorny tip (Fig. 6M, N). Both arm spines similar in size at distal end of arm, and turning into compound hook with 4–5 secondary teeth (Fig. 6O, P).

***Color*.** In live specimen, reddish brown (Fig. 6).

####### Ossicle morphology of paratype.

**Figure 7.**
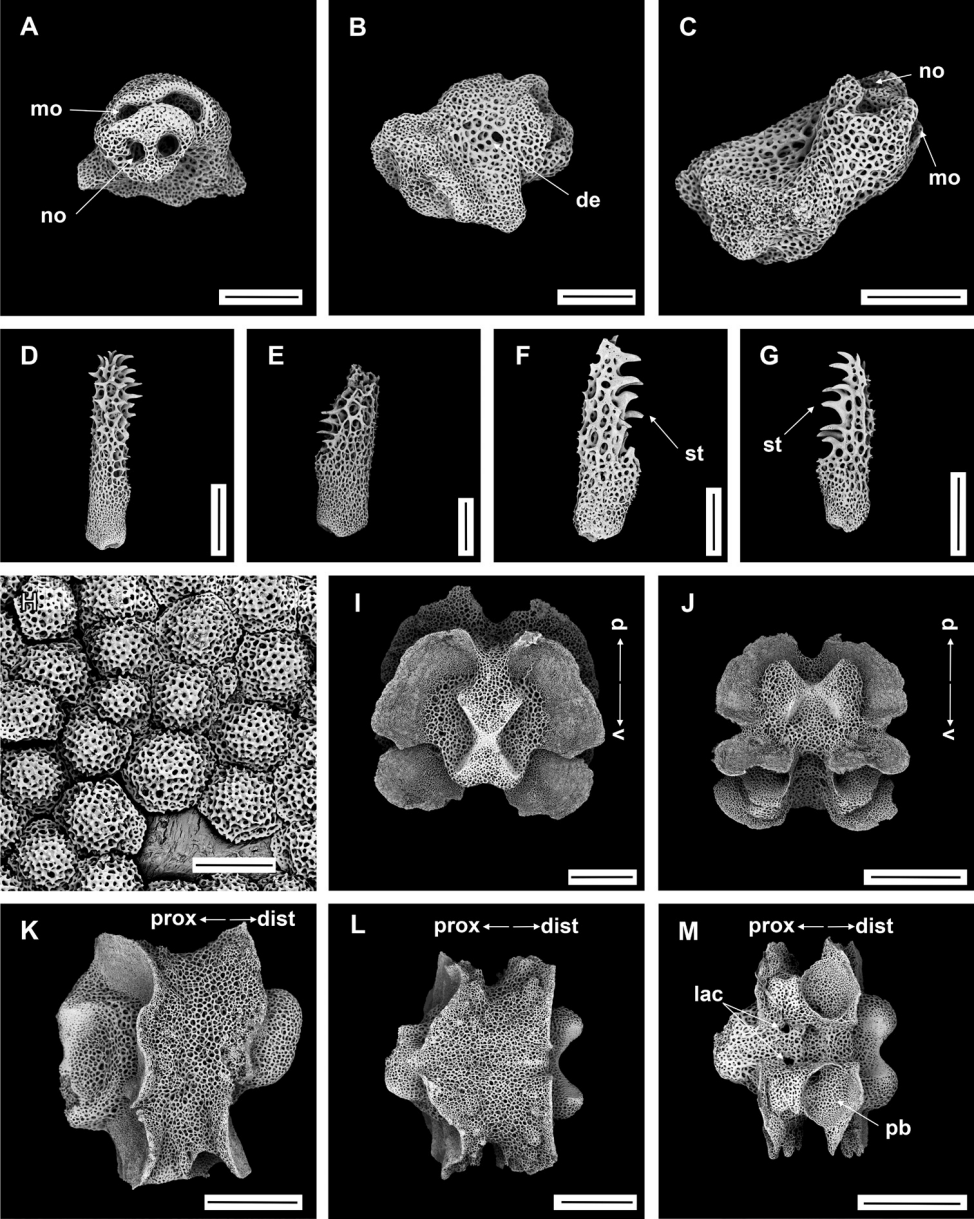
*Asteroschemashenhaiyongshii* sp. nov., paratype (IDSSE-EEB-SW0087) **A–C** lateral arm plate (external, internal) **D, E** arm spines (middle) **F, G** arm spine (distal) **H** skin from dorsal arm base (rounded granular ossicles) **I–M** vertebrae **I** proximal view **J** distal view **K** lateral view **L** dorsal view **M** ventral view. Abbreviations: **d** dorsal, **de** depression, **dist** distal, **lac** lateral ambulacral canals, **mo** muscle opening, **no** nerve opening, **pb** podial basin, **prox** proximal, **st** secondary teeth, **v** ventral. Scale bars: 500 µm (**D, I–M**); 300 µm (**A–C, E, G**); 200 µm (**F, H**).

Lateral arm plate curved around the vertebrae, with two arm spine articular structures, with completely separate large muscle and nerve openings (Fig. 7A). A depression on inner side of lateral arm plate (Fig. 5B, C). Inner arm spine becoming cylindrical from proximal to middle half of arm, with terminal projection, and thorny surface on upper part of spine (Fig. 7D). Outer arm spine nearly half as long as inner one with less thorny tip (Fig. 7E). Distally both spines compound hook with 3–5 secondary teeth (Fig. 7F, G). Arm and disc concealed by rounded granular ossicles (Fig. 7H). Vertebrae with streptospondylous articulation, with deep slope between proximal and distal end, dorsally a median longitudinal furrow, ventrally with median deep longitudinal groove with lateral ambulacral canals, no oral bridge, podial basins moderate in size (Fig. 7I–M).

####### Paratype variations.

Paratype disc diameter 12 mm, similar to holotype. Second arm spine from segments 10–12 and disc slightly flatter than those of holotype; considered intraspecific variation.

####### Distribution and habitat.

1070–1111 m depth. Near Xisha and Zhongsha islands in the South China Sea. Attached to sponge host.

####### Etymology.

The specific name is dedicated to the manned submersible vessel ‘Shenhaiyongshi’ meaning deep sea warrior in Chinese, which collected the specimen.

####### Remarks.

*Asteroschemashenhaiyongshii* sp. nov. concurs with the group of *Asteroschema* that has only granular ossicles. This group includes nearly 20 species. Furthermore, they are divided by naked, widely separated granular ossicles on ventral disc and base of arm. Fifteen *Asteroschema* species have dense granular ossicles on the ventral disc. *Asteroschemashenhaiyongshii* sp. nov. is distinguished clearly by its unique dense, rounded, fine granular ossicles on both disc and arms, a thick sub-cylindrical proximal to middle half of the arms without annular bands, and cylindrical outer arm spine with visible thorns at middle arm segments (Fig. 7).

*Asteroschemaajax* and *A.laeve* (Lyman, 1872) are similar to *A.shenhaiyongshii* sp. nov. by having granular ossicles on both dorsal and ventral disc, but are easily differentiated by well-spaced annulated granular bands on the arms, and club-shaped inner arm spine (Clark A. H. 1949; Lyman 1875; FWRI 2010).

*Asteroschemaarenosum* Lyman, 1878 is similar to *A.shenhaiyongshii* sp. nov. by having granular ossicles on both dorsal and ventral disc, but differs in having a swollen arm spine, sparser granular coverage (five grains in 1 mm), and a second arm spine from the fourth arm segment. *Asteroschemavicinum* Koehler, 1907 differs in its sub-equal arm spines and *A.sulcatum* Ljungman, 1872 in its highly dense granular ossicle coverage (9–15 grains in 1 mm). *Asteroschemaigloo* and *A.domogranulatum* sp. nov. differ from *A.shenhaiyongshii* sp. nov. by large polygonal to domed granular ossicles (four or five grains in 1 mm; Table 2) (Ljungman 1872; Lyman 1878; Koehler 1907; Pawson et al. 2009).

*Asteroschemaglaucum* Matsumoto, 1915, and *A.hemigymnum* Matsumoto, 1915 are similar to *A.shenhaiyongshii* sp. nov. by having granular ossicles on both dorsal and ventral disc, but differ by sparser granular ossicle coverage (five or six grains in 1 mm), club-shaped inner arm spine at the middle arm segments and in *A.hemigymnum* the ventral disc is covered with pavement-like ossicles. *Asteroschemainoratum* Koehler, 1906 is similar to *A.shenhaiyongshii* sp. nov. by having a similar density of the granular coverage on both dorsal and ventral disc, but differs in having granular ossicles on the ventral surface along the arm, a slightly club-shaped inner arm spine at middle arm segments, and both spines not transforming into a compound hook (Table 2) (Koehler 1906; Matsumoto 1915).

*Asteroschemamonobactrum* H. L. Clark, 1917 is similar to *A.shenhaiyongshii* sp. nov. in having granular ossicles on both dorsal and ventral surface and in start of the second arm spine, but differs by having separate, sparser granular ossicles (seven grains in 1 mm at disc center, five or six grains in 1 mm at periphery of disc and on arm), and by the granular ossicles on both sides distalwards along the arm becoming widely separated to almost naked (H. L. Clark 1917). *Asteroschemabrachiatum* Lyman, 1879 is similar to *A.shenhaiyongshii* sp. nov. in having similar density granular ossicle coverage on both dorsal and ventral disc and arms, but differs by the entire arm being covered by granular ossicles, start of the second arm spine at the fourth arm segment, and a slightly club-shaped inner spine at middle arm segments (Lyman 1879). *Asteroschemasalix* Lyman, 1879, *A.tubiferum* Matsumoto, 1911, and *A.rubrum* Lyman, 1879 differ from *A.shenhaiyongshii* sp. nov. by having separated, sparser granular ossicle coverage, a club-shaped inner spine at middle arm segments, and widely spaced ossicles on the ventral arm surface (Lyman 1879; Matsumoto 1911).

Granular ossicle density of *Asteroschemawrighti* McKnight, 2000, *A.bidwillae* McKnight, 2000, and *A.tenue* (eight or ten grains in 1 mm) is similar to *A.shenhaiyongshii* sp. nov. However, *Asteroschematenue* differs from *A.shenhaiyongshii* sp. nov. by having a club-shaped inner spine at middle arm segments, and slender long arms, and *A.wrighti* differs by having a club-shaped inner spine at middle arm segments, widely spaced granular ossicles on the ventral arm, irregular plate-like ossicles on the ventral disc, and smooth outer spines, and by being hexamerous (Table 2) (Lyman 1875; McKnight 2000).

Most *Ophiocreas* species differ from *Asteroschemashenhaiyongshii* sp. nov. by having naked or micro-granular ossicles in the skin in both disc and arms. (Table 2). In our phylogenetic tree of the family Euryalidae, all *Ophiocreas* species cluster with some *Asteroschema* species, but the average genetic distance between *Ophiocreas* species and *Asteroschemashenhaiyongshii* sp. nov. was 13.61% (Fig. 2, Suppl. material 2: Table S2). *Asteroschemashenhaiyongshii* sp. nov. clusters among *Asteroschemarubrum*, *A.salix*, *A.tubiferum*, A.cf.lissum, and *A.bidwillae*. All these *Asteroschema* species have granular ossicles on the dorsal disc and dorsal surface of the arms. *Asteroschemabidwillae* showed a close relationship with *A.shenhaiyongshii* sp. nov., and was identified as a sibling species due to similar morphological characters of granular ossicles on the dorsal disc and dorsal surface of the arms, and the shape of the inner arm spines at middle arm segments, but differs by having well-spaced granular ossicles on the ventral disc, a naked proximal ventral arm surface, and by being hexamerous and fissiparous (Table 2, Suppl. material 2: Table S2).

###### ﻿ Asteroschema cf. bidwillae

Taxon classificationAnimaliaEuryalidaEuryalidae

McKnight, 2000

D2E2CB29-5574-57E2-8F3A-F1CB6054926A

Figures 8 , 9

Asteroschema bidwillae McKnight, 2000: 24–27, fig. 8.

####### Material examined.

China • 1 specimen; South China Sea, Zhongsha Islands, seamount; 13°36.20'N, 113°33.74'E; depth 1515 m; 30 Mar. 2020; Collecting event: stn. SC025; ‘Shenhaiyongshi’ msv leg; preserved in -80 °C; IDSSE-EEB-SW0105.

####### Description.

**Figure 8.**
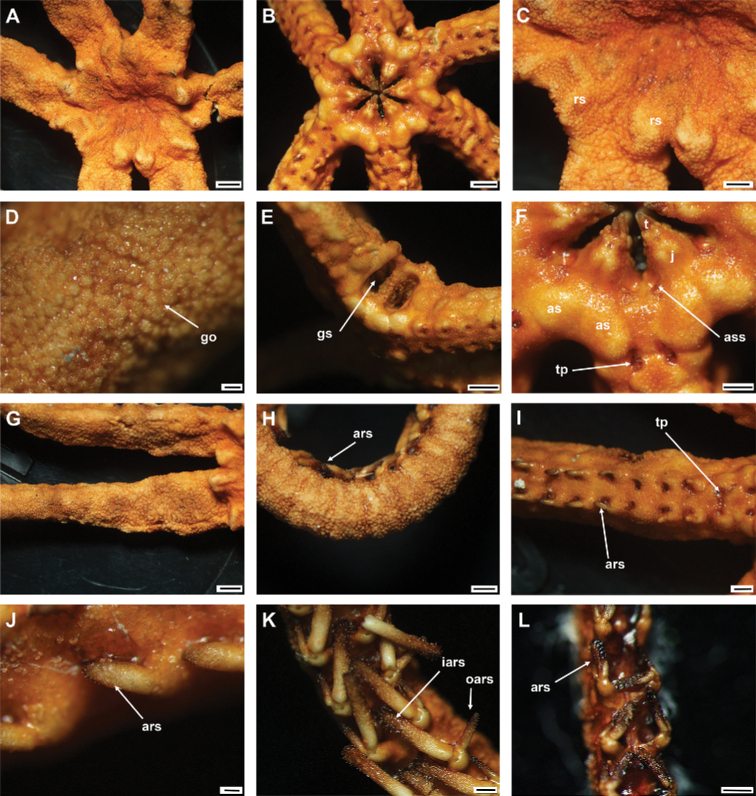
Asteroschemacf.bidwillae McKnight, 2000 (IDSSE-EEB-SW0105) **A** dorsal side of the specimen **B** ventral side of the specimen **C** dorsal disc **D** skin on the disc **E** lateral disc **F** oral frame **G** dorsal arms (proximal) **H** dorsal arm (middle) **I** ventral arm (arm base) **J** outer arm spine (proximal) **K** arm spines (middle) **L** arm spines (distal). Abbreviations: **ars** arm spine, **as** adoral shield, **ass** adoral shield spine, **go** granular ossicle, **gs** genital slit, **iars** inner arm spine, **j** jaw, **oars** outer arm spine, **rs** radial shield, **t** teeth, **tp** tentacle pore. Scale bars: 2 mm (**A–C, E, G**); 1 mm (**F, H, I**); 500 µm (**K, L**); 200 µm (**D, G, J**).

Disc diameter 13 mm, length of arms 195 mm, arm base width 4.5–5 mm (Fig. 8).

***Disc*.** Disc circular, hexamerous, raised above arms, deeply swollen in center (Fig. 8A, B). Disc covered with dense, small, finely rugose granular epidermal ossicles (Fig. 8A–C). Granular ossicles dense and small in size in disc center, but slightly larger at distal edge (six or seven grains in 1 mm; Fig. 8C, D). Radial shields extending to center but proximal ends concealed by skin with granular ossicles, and distal ends raised above the disc (Fig. 8C). Granular ossicles around distal edge and periphery of disc larger and more irregular (Fig. 8C). Genital slits narrow, without ossicles and vertical on ventral interradii (Fig. 8E). Jaws elongated, mostly naked without granular ossicles (Fig. 8F). At apex of jaw flattened, pointed, and finely rugose teeth, and two to four granular tubercles that resemble lateral oral papillae (Fig. 8F). Ventral disc covered with widely separated small granular ossicles (four or six grains in 1 mm; Fig. 8B, F). Adoral shields connected to first ventral arm segment and concealed by widely separated small granular ossicles, but outline of shields clearly visible (Fig. 8F). Oral shields not discernible and adoral shield spine covered by ossicles (Fig. 8F).

***Arms*.** Arms six, at base wide, not arched, dorsally flattened, and swollen in first few free arm segment (Fig. 8G). Arms distalwards from middle part narrowing and increasingly cylindrical (Fig. 8H). Swollen dorsal arm base covered with dense, large, irregular granular ossicles (four or seven grains in 1 mm), distalwards decreasing in size and becoming rounded (six or seven grains in 1 mm), and distally widely separated (Fig. 8G, H). Lateral arm plates covered with slightly separated granular ossicles. Ventral arm near base covered with granular ossicles similar to ventral disc (five or six grains in 1 mm), but becoming widely separated to completely naked along the arm (Fig. 8I, J). First two or three arm segments without arm spine (Fig. 8I). First arm spine appeared at third or fourth arm segment, and second arm spine at eighteenth or twenty-first segment (Fig. 8I–K). Inner arm spine cylindrical, with blunt thorny tip, one and a half arm segment in length (Fig. 8J, K). Outer spine half as long as inner spine in middle region, with thorny, pointed tip (Fig. 8K). Both arm spines equal in length at distal end of arm, and compound hook with 3–6 secondary teeth (Fig. 8L).

***Color*.** In live specimen, reddish brown (Fig. 8).

####### Ossicle morphology.

**Figure 9.**
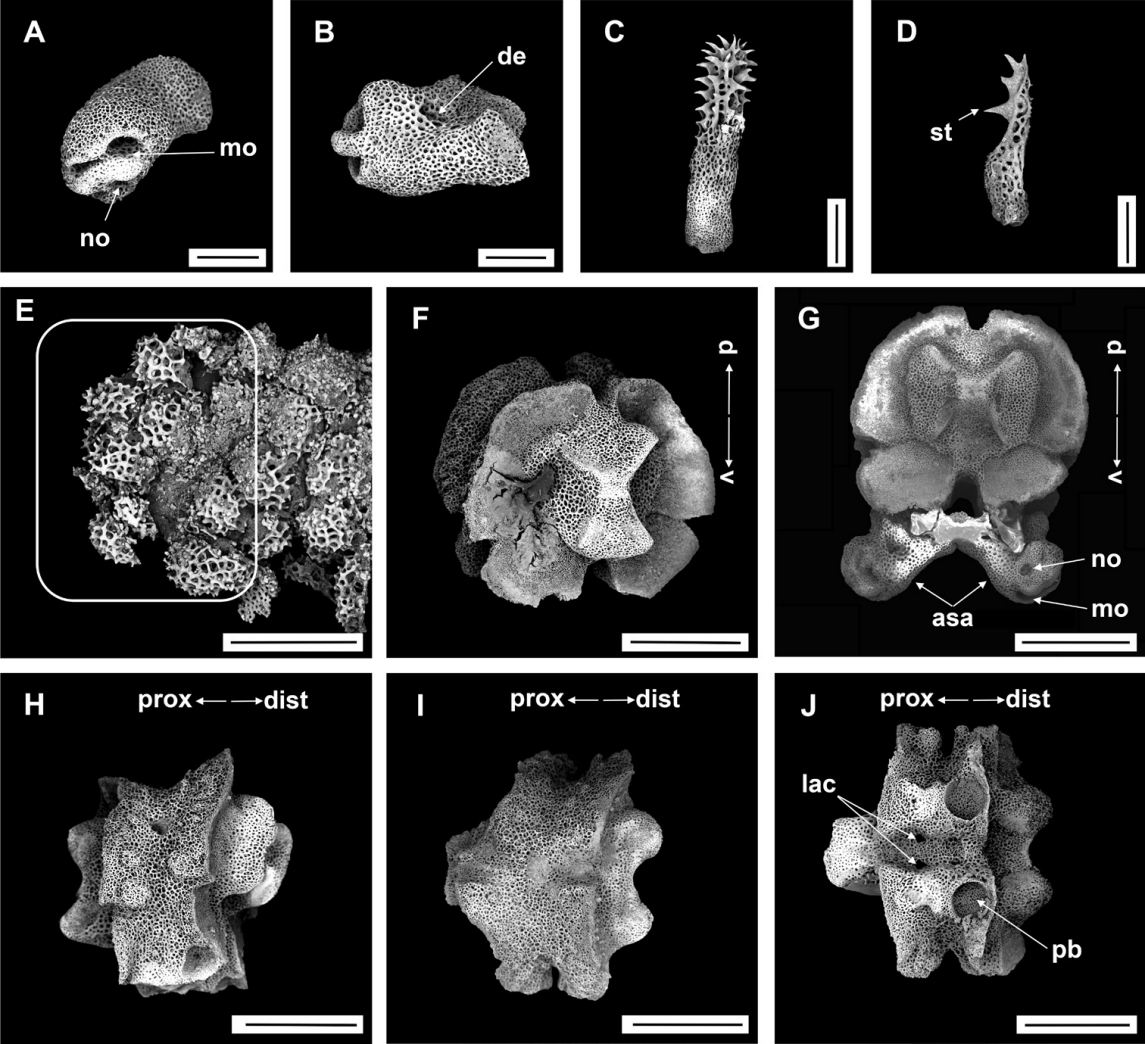
Asteroschemacf.bidwillae McKnight, 2000 (IDSSE-EEB-SW0105) **A, B** lateral arm plate (external, internal) **C** outer arm spine (middle) **D** arm spine (distal) **E** skin from dorsal arm base (rounded to irregular-shaped granular ossicles) **F–J** vertebrae **F** proximal view **G** distal view **H** lateral view **I** dorsal view **J** ventral view. Abbreviations: **asa** arm spine articulation, **d** dorsal, **de** depression, **dist** distal, **lac** lateral ambulacral canals, **mo** muscle opening, **no** nerve opening, **pb** podial basin, **prox** proximal, **st** secondary teeth, **v** ventral. Scale bars: 800 µm (**F–J**); 500 µm (**C**); 300 µm (**A, B, E**); 200 µm (**D**).

Lateral arm plate with two arm spine articular structures, with slightly separated large muscle and relatively small nerve opening (Fig. 9A). A depression on inner side of lateral arm plate (Fig. 9B). Inner arm spine from proximal and middle half of arm with cylindrical, terminal projection, and upper part of spine covered with thorns (Fig. 9C). Distally, arm spine turns into compound hook with secondary teeth (Fig. 9D). Arm and disc concealed by rounded to slightly irregular granular ossicles (Fig. 9E). Vertebrae with streptospondylous articulation, dorsally a median large longitudinal furrow, ventrally with median deep groove with lateral ambulacral canals, podial basins small (Fig. 9F–J).

####### Distribution.

400–2000 m depth. New Zealand, Tasman Sea, Solomon Islands, South China Sea (OBIS 2021).

####### Remarks.

*Asteroschemabidwillae* was first described by McKnight (2000), with type locality New Zealand waters in the South Pacific Ocean. This is the first redescription since the original description. The specimens from our collection concur with McKnight’s description, but we noticed some differences such as: granular arrangement on radial shields, irregular ossicles on arm base, ossicles on ventral arm recorded nearly to middle region, and start of second arm spine. However, some of these variations may be related to size and maturity of the specimen (the holotype had a disc diameter of 5 mm). We hesitate to fully associate our specimen with *Asteroschemabidwillae* due to uncertainty with the morphological variation in *A.bidwillae*. The genus *Asteroschema* contains only two hexamerous species as far as known. Therefore, the closest one is *Asteroschemawrighti* McKnight, 2000, but it differs in characters of the radial shields, granulation on disc and arm, innermost arm spine, and start of second arm spine (Table 2). This is the first record of *A.bidwillae* from the North Pacific Ocean, if it is indeed this species.

###### ﻿ Asteroschema rubrum

Taxon classificationAnimaliaEuryalidaEuryalidae

Lyman, 1879

42A26B36-728D-5797-8383-B380FCAE07A5

Figures 10 , 11

Asteroschema rubrum Lyman, 1879: 68–69, fig. 17, figs 454–457.

####### Material examined.

China • 3 specimens; South China Sea, near Zhongsha Islands, seamount; 13°55.44'N, 115°25.37'E; depth 958 m; 09 Mar. 2020; Collecting event: stn. SC007; ‘Shenhaiyongshi’ msv leg; preserved in -80 °C; GenBank: OK044293, OL712209, OK044294, OL712210, OK044295, OL712211; IDSSE-EEB-SW0071, IDSSE-EEB-SW0072, IDSSE-EEB-SW0073 • 1 specimen; South China Sea, near Zhongsha Islands, seamount; 14°21.93'N, 115°23.89'E; depth 922 m; 17 Mar. 2020; Collecting event: stn. SC035; ‘Shenhaiyongshi’ msv leg; preserved in -80 °C; IDSSE-EEB-SW0088.

####### Description.

**Figure 10.**
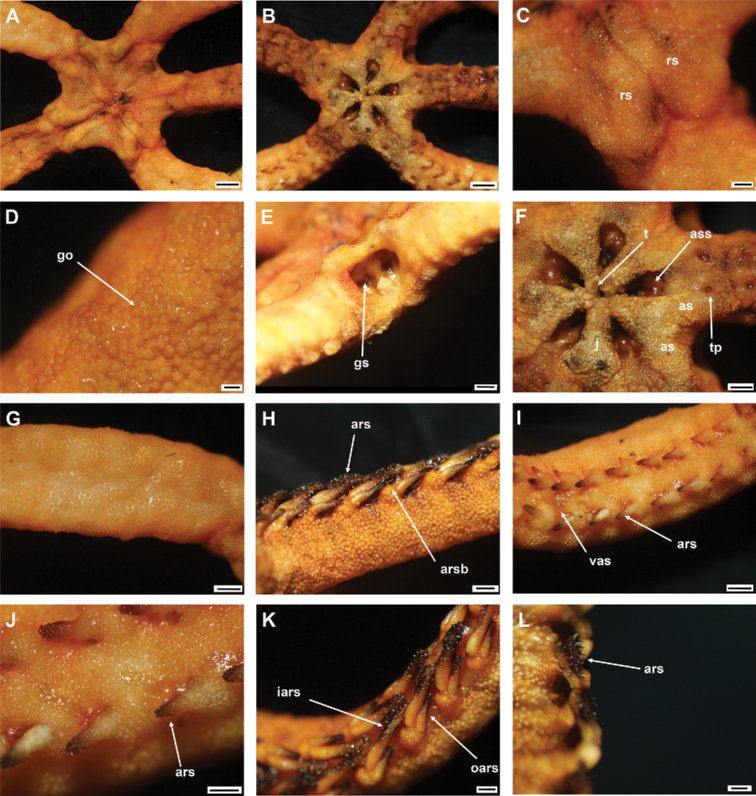
*Asteroschemarubrum* Lyman, 1879 (IDSSE-EEB-SW0072) **A** dorsal view **B** ventral view **C** dorsal disc **D** skin on the disc **E** lateral disc **F** ventral disc **G** dorsal arm (proximal) **H** lateral arm (middle) **I** ventral arm (proximal) **J** outer arm spine (proximal) **K** arm spines (middle) **L** arm spines (distal). Abbreviations: **as** adoral shield, **ass** adoral shield spine, **ars** arm spine, **go** granular ossicle, **gs** genital slit, **iars** inner arm spine, **j** jaw, **oars** outer arm spine, **ass** adoral shield spine, **rs** radial shield, **t** teeth, **tp** tentacle pore. Scale bars: 2 mm (**A, B**); 1 mm (**E–G, I**); 500 µm (**C, H, J, K**); 200 µm (**D, L**).

IDSSE-EEB-SW0072: disc diameter 12 mm, length of arms from 165–175 mm (Fig. 10).

***Disc*.** Disc flat, slightly raised above arms, swollen in center, and small in relation to total body size of specimen (Fig. 10A, B). Disc covered with smooth, dense, evenly distributed, small rounded or irregular granular ossicles, similar in size (seven or eight granular ossicles in 1 mm; Fig. 10C, D). Radial shields closely together, parallel, raised above distal disc edge, but mostly concealed by skin with granular ossicles (Fig. 10C). Radial shields do not meet in center. Genital slits wide and vertical on ventral interradii (Fig. 10E). Jaw large, long and covered with dense irregular ossicles (Fig. 10F). Spearhead-shaped teeth and granular ossicles that resemble lateral oral papillae at apex of jaw (Fig. 10F). Adoral shields large, connected to first ventral arm segment, and concealed by granular ossicles (Fig. 10F). Oral shields not discernible and adoral shield spine naked. Whole oral region swollen nearly to genital slit. Ventral disc covered with dense granular ossicles (seven or eight grains in 1 mm; Fig. 10F).

***Arms*.** Arms at base wide, not arched, dorsally flattened, and slightly swollen in first few free segments (Fig. 10A, G). Arms distalwards from middle part narrowing and more cylindrical (Fig. 10H). Dorsal arm base covered with smooth rounded granular ossicles (six or seven grains in 1 mm), middle segments with dense granular ossicles all the way to the arm spine base (seven or eight grains in 1 mm), and distally decreasing in size and separated (seven or eight grains in 1 mm) (Fig. 10G–L). Ventral arm base covered with dense granular ossicles similar to the ventral disc (eight or nine grains in 1 mm), distally decreasing in size and separated to naked (Fig. 10H–K). First one to two tentacle pores without arm spine (Fig. 10F). First arm spine appears at second or third arm segment, and second arm spine at ninth or eleventh segment (Fig. 10I). Outer arm spine half as long as inner spine in middle region, thorny pointed tip, distally compound hook (Fig. 10K, L). Inner arm spine cylindrical, one to one and a half arm segment in length, initially tapering to a pointed thorny tip, in middle blunt, slightly swollen with thorny surface on more than half its length, distally compound hook with three or four secondary teeth (Fig. 10I–L).

***Color*.** In live specimen, reddish brown (Fig. 10).

####### Ossicle morphology.

**Figure 11.**
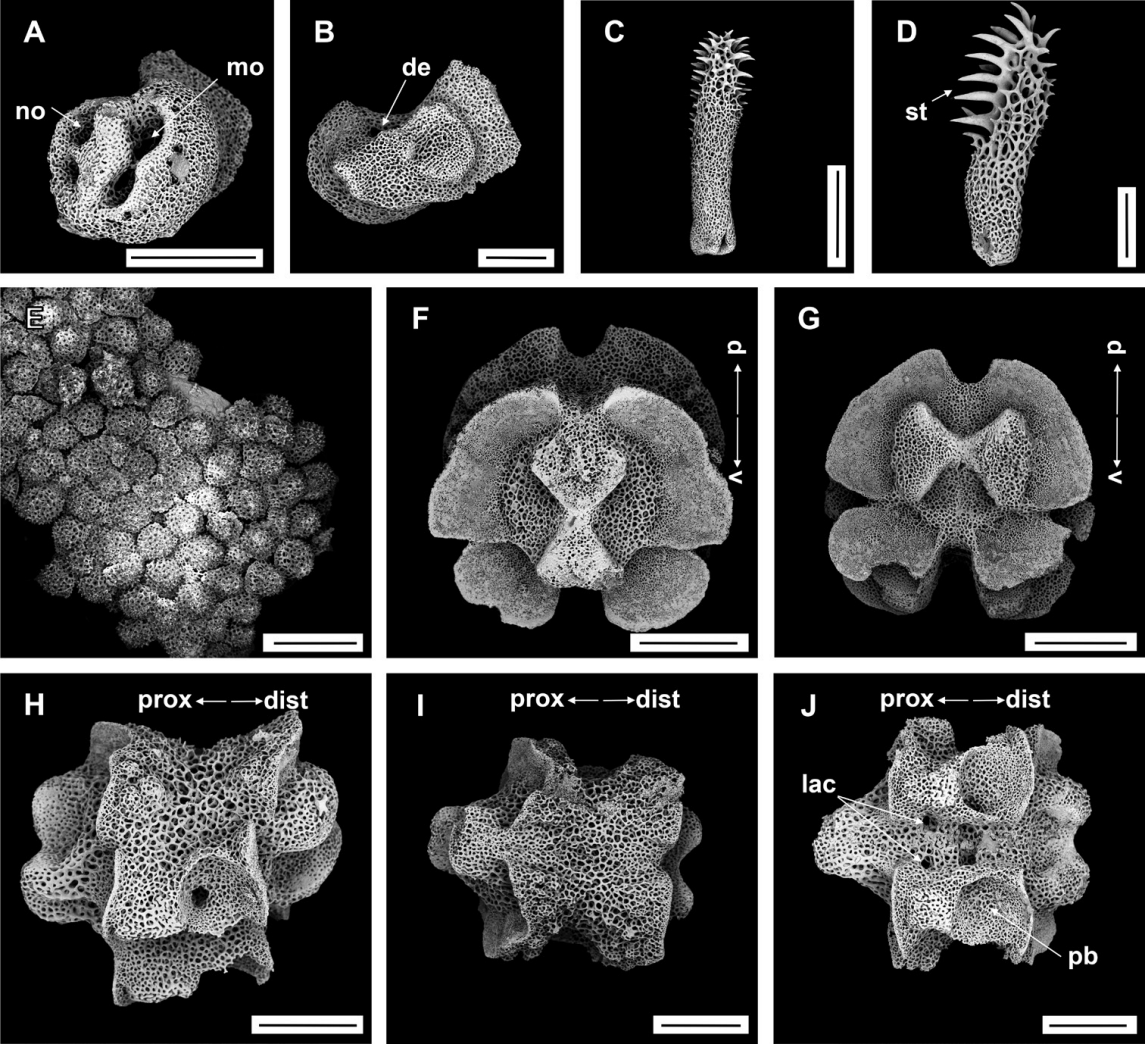
*Asteroschemarubrum* Lyman, 1879 (IDSSE-EEB-SW0072) **A, B** lateral arm plate (external, internal), **C** outer arm spine (middle), **D** arm spine (distal), **E** skin from dorsal arm base (rounded to somewhat cone shaped granular ossicles), **F–J** vertebrae; **F** proximal view, **G** distal view, **H** lateral view, **I** dorsal view, **J** ventral view. Abbreviations: **d** dorsal, **de** depression, **dist** distal, **lac** lateral ambulacral canals, **mo** muscle opening, **no** nerve opening, **pb** podial basin, **prox** proximal, **st** secondary teeth, **v** ventral. Scale bars: 800 µm (**C**); 500 µm (**A, F, G**); 300 µm (**B, E, H–J**); 200 µm (**D**).

Lateral arm plate with two arm spine articular structures, with slightly separate large muscle and nerve openings (Fig. 11A). A depression on inner side of lateral arm plate (Fig. 11B). Inner arm spine from proximal and middle half of arm cylindrical, slightly swollen, with thorny tip (Fig. 11C). Distally arm spine turns into compound hook with secondary teeth (Fig. 11D). Arm and disc concealed by granular ossicles, slightly wider than high, round to short stumps with convex tip (Fig. 11E). Vertebrae with streptospondylous articulation, with deep slope between proximal and distal end, dorsally a median longitudinal groove, ventrally with median deep longitudinal groove with lateral ambulacral canals, podial basins moderate in size (Fig. 11F–J).

####### Distribution.

730–958 m depth. Near Brandella, Chile and in the South China Sea.

####### Remarks.

*Asteroschemarubrum* was first described by Lyman (1879), with type locality in the Southwest Pacific Ocean near South America. This is the first rediscovery after the original description. The specimens from our collection concur well with Lyman’s holotype description, the only difference was the starting point of the second arm spines. However, this morphological character is highly variable among individuals. According to the holotype description, *A.rubrum* belongs in the clade with granular ossicles only in the genus *Asteroschema*, but Okanishi and Fujita (2009) considered *A.rubrum* in the clade with conical and granular ossicles. Although, the SEM images of granular ossicles in the skin appear as granular to somewhat small stumps with convex tip (Fig. 11E). However, the description of the holotype is identical with our specimen, and it was described as granular ossicles. The characters of the swollen oral region, smooth granulation on the disc, and innermost spine can be used to distinguish *A.rubrum* from other species of *Asteroschema* (Table 2). The dorsal disc of *A.rubrum* seems naked in wet condition due to its smooth granulation which can lead to misidentification as *Ophiocreas* species (Fig. 10A). This is the first record from the South China Sea.

###### ﻿ Asteroschema tubiferum

Taxon classificationAnimaliaEuryalidaEuryalidae

Matsumoto, 1911

65DB2915-3978-5AAC-A2D4-2F51DDAE884D

Figures 12 , 13

Asteroschema tubiferum Matsumoto, 1911: 52; 1917: 44; Baker 1980: 22, fig. 4; McKnight 2000: 24, fig. 7.

####### Material examined.

China • 1 specimen; South China Sea, Zhongsha Islands, seamount; 13°36.20'N, 113°33.74'E; depth 1515 m; 30 Mar. 2020; Collecting event: stn. SC025; ‘Shenhaiyongshi’ msv leg; preserved in -80 °C; GenBank: OK044298; IDSSE-EEB-SW0077 • 1 specimen; South China Sea, East of Zhongsha Islands, seamount; 16°22.11'N, 116°06.60'E; depth 1619 m; 09 Aug. 2020; Collecting event: stn. SC028; ‘Shenhaiyongshi’ msv leg; preserved in -80 °C; GenBank: OK044297, OL712213; IDSSE-EEB-SW0106. Northwest Pacific • 1 specimen; near Mariana Trench, Southeast of Guam Island, deepsea seamount, 11°57.20'N, 141°28.67'E; depth 1377 m; 03 Sep. 2019; Collecting event: stn. SC034; ‘Shenhaiyongshi’ msv leg; preserved in -80 °C; GenBank: OK044296, OL712212; IDSSE-EEB-SW0078.

####### Description.

**Figure 12.**
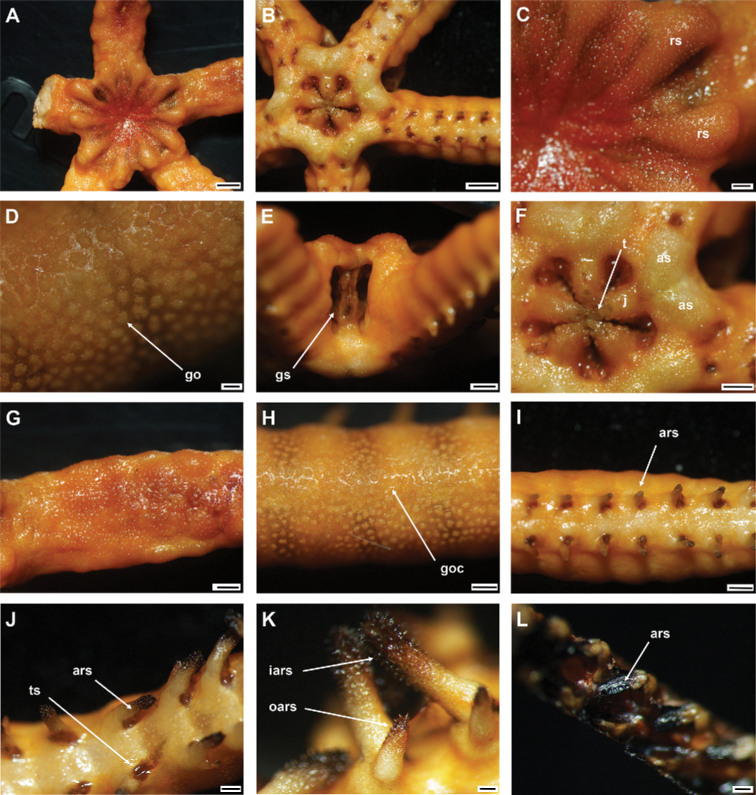
*Asteroschematubiferum* Matsumoto, 1911 (IDSSE-EEB-SW0078) **A** dorsal view **B** ventral view **C** dorsal disc **D** skin on the arm **E** lateral disc **F** ventral disc **G** dorsal arm (proximal) **H** dorsal arm (middle) **I** ventral arm (proximal) **J** outer arm spine (proximal) **K** arm spines (middle) **L** arm spines (distal). Abbreviations: **as** adoral shield, **ars** arm spine, **go** granular ossicle, **gs** genital slit, **iars** inner arm spine, **j** jaw, **oars** outer arm spine, **rs** radial shield, **t** teeth, **tp** tentacle pore. Scale bars: 2 mm (**A, B**); 1 mm (**E–G, I**); 500 µm (**C, H, J, K**); 200 µm (**D, L**).

IDSSE-EEB-SW0078: disc diameter 10 mm, length of arms 200 mm (Fig. 12).

***Disc*.** Disc flat, slightly raised above arms, swollen in center (Fig. 12A, B). Disc covered with smooth, small, closely spaced, and evenly rounded or polygonal granular ossicles, dense in disc center (seven or eight grains in 1 mm), but larger and polygonal at distal edge (six or seven grains in 1 mm) (Fig. 12C, D). Radial shields not meeting in center, but converging (Fig. 12C). Distal end of radial shields raised above disc and wider than proximal end (Fig. 12C). Genital slits narrow, vertical on ventral interradii (Fig. 12E). Jaws elongated, covered densely with granular ossicles (Fig. 12F). Flattened, pointed, and spearhead-shaped teeth and granular ossicles that resemble lateral oral papillae at apex of jaw (Fig. 12F). Ventral disc densely covered with granular and polygonal ossicles (seven or eight grains in 1 mm; Fig. 12F). Adoral shields connected to first ventral arm segment and concealed by granular ossicles but outline of shields visible. Oral shields not discernible and adoral shield spine densely covered by ossicles (Fig. 12F).

***Arms*.** Arms at base wide, dorsally flattened, and swollen in first few free segments (Fig. 12G). Arms distalwards from middle part narrowing and more cylindrical (Fig. 10H). Swollen dorsal arm base and proximal end of arm covered with dense granular or polygonal ossicles similar to disc (five or seven grains in 1 mm), on middle segments with slightly separated granular ossicles (six or seven grains in 1 mm), and distally decreasing in size and separated (seven or eight grains in 1 mm) (Fig. 12G, H). Granular ossicles on lateral arm plates slightly separated, but continuing along arm. Ventral arm near arm base covered with granular ossicles similar to ventral disc (seven or eight grains in 1 mm), but less dense on middle half (five or six grains in 1 mm), and distally widely separated or naked (six or seven grains in 1 mm) (Fig. 12I, J). On first few arm segments, tentacle pore with extended tube (Fig. 12J). First tentacle pore without arm spine (Fig. 12F). First arm spine appears at second arm segment, and second arm spine at eighth segment. Inner arm spine cylindrical, one arm segment in length, with blunt thorny tip, and slightly club-shaped (Fig. 12K). Outer arm spine smaller in size, with smooth to thorny tip (Fig. 12K). Both arm spines equal in length at distal end of arm, and compound hook with three or four secondary teeth (Fig. 12L).

***Color*.** In live specimen, reddish brown on dorsal disc and arm, light brown on ventral disc and arm (Fig. 12).

####### Ossicle morphology.

**Figure 13.**
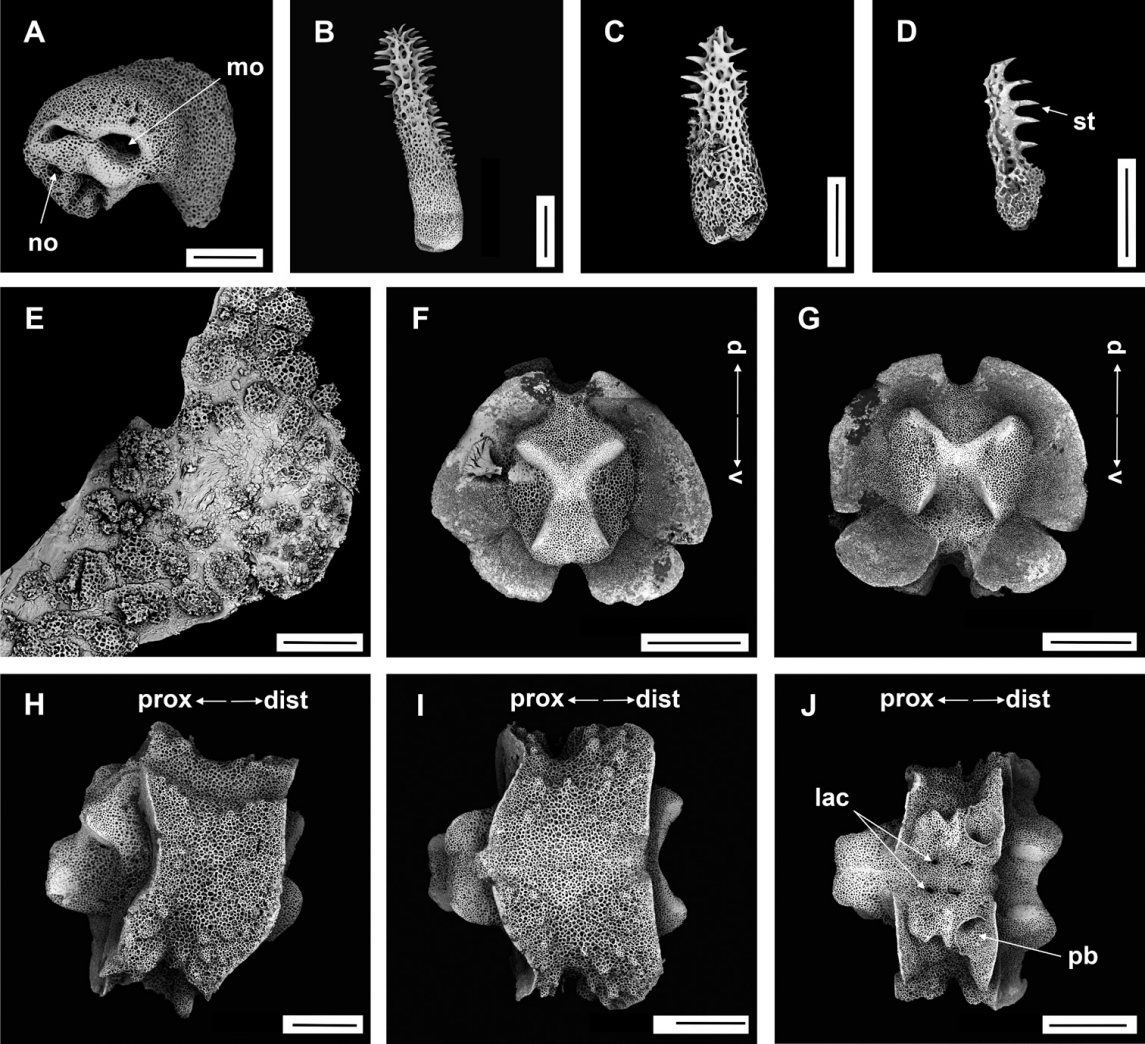
*Asteroschematubiferum* Matsumoto, 1911 (IDSSE-EEB-SW0078) **A** lateral arm plate **B** outer arm spine (middle) **C** inner arm spine (middle) **D** arm spine (distal) **E** skin from dorsal arm base (granular ossicles) **F–J** vertebrae **F** proximal view **G** distal view **H** lateral view **I** dorsal view **J** ventral view. Abbreviations: **d** dorsal, **dist** distal, **lac** lateral ambulacral canals, **mo** muscle opening, **no** nerve opening, **pb** podial basin, **prox** proximal, **st** secondary teeth, **v** ventral. Scale bars: 800 µm (**F, G, J**): 500 µm (**B, H, I**); 300 µm (**A, C–E**).

Lateral arm plate with two arm spine articular structures, with large, separated muscle and nerve openings (Fig. 13A). Inner arm spine distalwards from proximal part of arm cylindrical, with terminal projection, and thorny surface (Fig. 13B). Outer arm spine cylindrical, with pointed tip with few thorns (Fig. 13C). Distally arm spine turns into compound hook with four secondary teeth (Fig. 13D). Arm and disc concealed by less dense, wider, and shorter granular ossicles (Fig. 13E). Vertebrae with streptospondylous articulation, dorsally a large longitudinal furrow, ventrally with deep median longitudinal groove with lateral ambulacral canals, no oral bridge, podial basins relatively small (Fig. 13F–J).

####### Distribution.

325–1800 m depth. New Zealand, Tasman Sea, Kermadec Islands, Bay of Plenty, Hawaii, Sagami Sea, the South China Sea, and Northwest Pacific seamount.

####### Remarks.

*Asteroschematubiferum* was first described by Matsumoto (1911), then redescribed by Matsumoto (1917), Baker (1980), and McKnight (2000). These redescriptions are helpful to identify individual morphological character variation. Matsumoto (1911, 1917) mentioned that in *Asteroschematubiferum* the first ten arm segments have an extended tube in the tentacle pore (a sheath around the tentacle) that is closely attached to the arm spine and this character is present in our specimens. Previously, *A.tubiferum* had been recorded from both North and South Pacific Oceans at a wide distribution range. However, this is the first record from the South China Sea. The specimens from our collection concur with previous redescriptions, but we noticed a few variations such as less densely packed ossicles on ventral and lateral arm. The *A.tubiferum* specimen from the South China Sea collection showed less dense granular ossicles on the ventral disc and arm. *Asteroschematubiferum* strongly resembles *A.rubrum*, *A.laeve*, and *A.inoratum*, but the characters of the granulation pattern, tentacle scale on first few arm segments, shape of the arm and inner arm spine characters can be used to distinguish it from these species (Table 2).

###### ﻿ Asteroschema salix

Taxon classificationAnimaliaEuryalidaEuryalidae

Lyman, 1879

6AA0A5B6-5BD5-53F3-BA59-D1CDDFD2CFBA

Figures 14 , 15

Asteroschema salix Lyman, 1879: 66–67, fig. 17, figs 466–469; 1882: 277, fig. 22, figs 13–15; Baker 1980: 22; McKnight 2000: 21–22, fig. 6; Olbers et al. 2015: 85, fig. 1A, B; 2019: 51–52, fig. 24–25.

####### Material examined.

China • 1 specimen; South China Sea, Zhongsha Islands, seamount; 15°36.64'N, 116°7.73'E; depth 1775 m; 19 Sep. 2020; Collecting event: stn. SC010; ‘Shenhaiyongshi’ msv leg; preserved in 95% ethanol; GenBank: OK044301, OL712214; IDSSE-EEB-SW0082.

####### Description.

Disc diameter 10 mm, length of arms 145 mm (Fig. 12).

**Figure 14.**
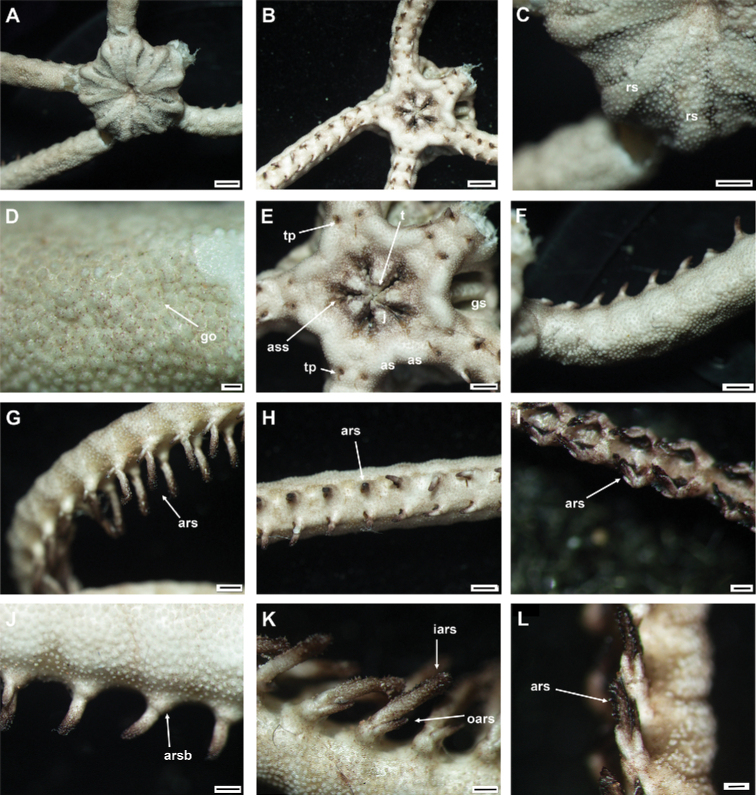
*Asteroschemasalix* Lyman, 1879 (IDSSE-EEB-SW0082) **A** dorsal view **B** ventral view **C** dorsal disc **D** skin on the arm **E** ventral disc **F** dorsal arm (proximal) **G** lateral arm (middle) **H** ventral arm (proximal) **I** ventral arm (distal) **J** outer arm spine (proximal) **K** arm spines (middle) **L** arm spines (distal). Abbreviations: **as** adoral shield, **ass** adoral shield spine, **ars** arm spine, **go** granular ossicle, **gs** genital slit, **iars** inner arm spine, **j** jaw, **oars** outer arm spine, **ots** oral tentacle scale, **rs** radial shield, **t** teeth, **tp** tentacle pore. Scale bars: 2 mm (**A, B**); 1 mm (**C, E–H**); 500 µm (**J, K**); 200 µm (**D, I, L**).

***Disc*.** Disc flat, strongly raised above arms (Fig. 14A, B). Disc covered by thin skin with fine, small, rounded granular ossicles, dense in center (seven or eight grains in 1 mm), but separated at distal edge (six or seven grains in 1 mm) (Fig. 14C, D). Radial shields long, narrow, widely separated distally, convergent proximally, meeting in disc center (Fig. 14C). Genital slits narrow, and vertical on ventral interradii (Fig. 14E). Jaws elongated, covered with granular ossicles but near apex fewer granular ossicles (Fig. 14E). Flattened, spearhead-shaped teeth and granular ossicles that resemble lateral oral papillae at apex of jaw (Fig. 14E). Ventral disc covered with granular ossicles similar to dorsal disc (six or seven grains in 1 mm), slightly separated (Fig. 14E). Adoral shields large, connected to first ventral arm segment, concealed by thin skin with granular ossicles, but plate outline visible (Fig. 14E). Oral shields not discernible and adoral shield spine covered with ossicles (Fig. 14E).

***Arms*.** Arms sub-cylindrical, not swollen, narrower and more cylindrical in distal half of arm (Fig. 14F, G). Dorsal and lateral arm base covered with granular ossicles similar to disc (six or seven grains in 1 mm), on middle segments granular coverage similar to arm base (six or seven grains in 1 mm), and distally decreasing in size and widely separated (grains six or eight in 1 mm) (Fig. 14F, G). On lateral arm plate, granular ossicles continue toward base of arm spine (Fig. 14G, J). Ventral surface of arm base covered with granular ossicles similar to ventral disc but less dense (six or seven grains in 1 mm), widely separated and decreasing in size to naked at middle to distal end of arm (Fig. 14H, I). First tentacle pore without arm spine (Fig. 14E). First arm spine appears at second arm segment, second arm spine at fifteenth or nineteenth segment. Inner arm spine cylindrical, one arm segment in length, flattened, with blunt, thorny tip, slightly club-shaped (Fig. 14J, K). Outer arm spine half as long as inner, with thorny tip (Fig. 14K). Both arm spines equal in length at distal end of arm, and turning into compound hook with 3–5 secondary teeth (Fig. 14L).

***Color*.** In ethanol, pink but when dried, dull brown to whitish (Fig. 14).

####### Ossicle morphology.

**Figure 15.**
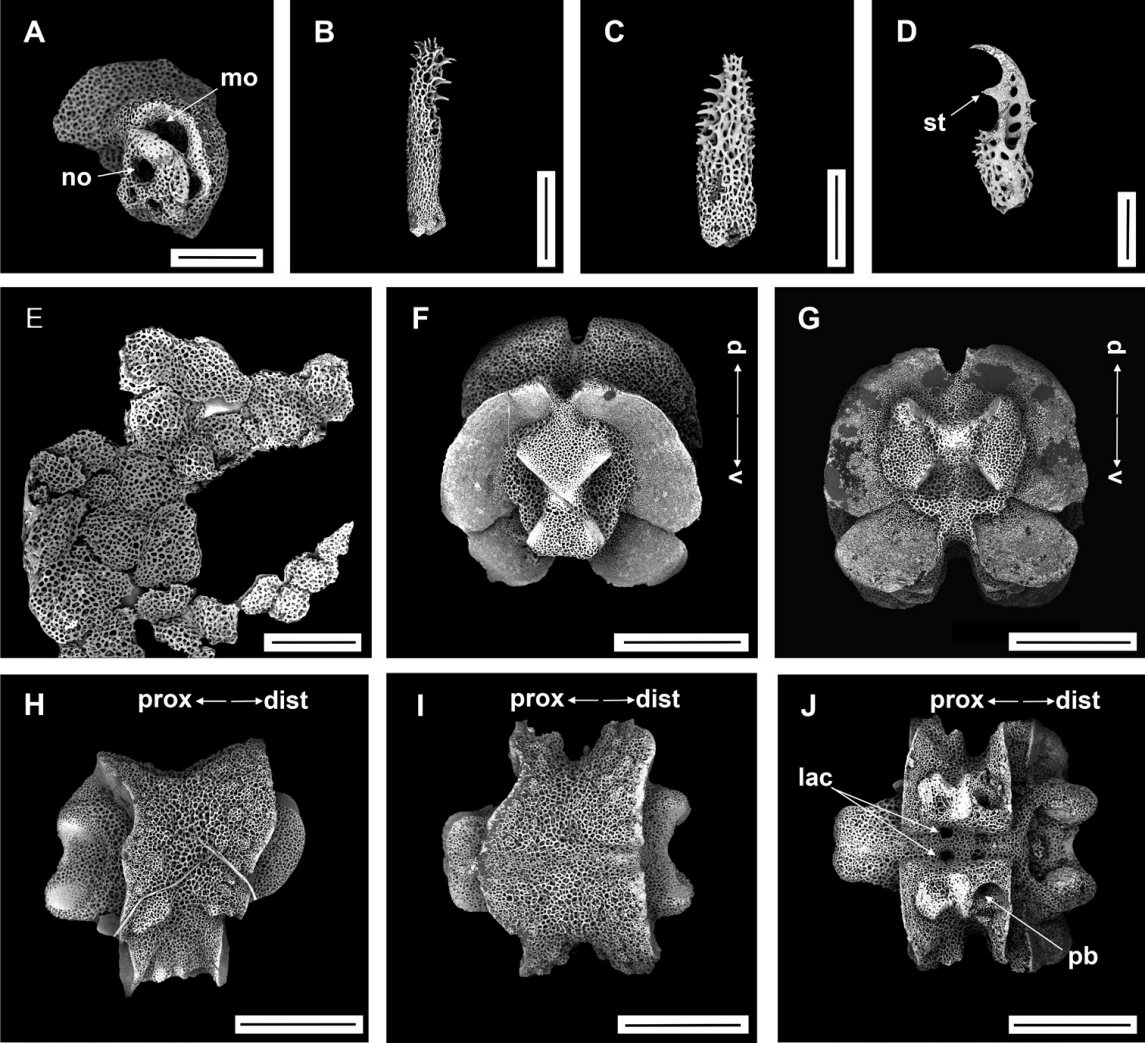
*Asteroschemasalix* Lyman, 1879 (IDSSE-EEB-SW0082) **A** lateral arm plate **B** outer arm spine (middle) **C** inner arm spine (middle) **D** arm spine (distal) **E** skin from dorsal arm base (granular ossicles) **F–J** vertebrae **F** proximal view **G** distal view **H** lateral view **I** dorsal view **J** ventral view. Abbreviations: **d** dorsal, **dist** distal, **lac** lateral ambulacral canals, **mo** muscle opening, **no** nerve opening, **pb** podial basin, **prox** proximal, **st** secondary teeth, **v** ventral. Scale bars: 800 µm (**B, F–J**); 300 µm (**A, C, E**); 100 µm (**D**).

Lateral arm plate with two arm spine articular structures, with two large muscle and nerve openings (Fig. 15A). Inner arm spine at proximal and middle half of arm cylindrical, with thorny tip (Fig. 15B). Outer arm spine cylindrical with pointed tip (Fig. 15C). Distally arm spine turns into compound hook with secondary teeth (Fig. 15D). Arm and disc concealed by wider polygonal to rounded granular ossicles (Fig. 15E). Vertebrae with streptospondylous articulation, dorsally a median longitudinal furrow, ventrally with deep median longitudinal groove with lateral ambulacral canals, no oral bridge, podial basins relatively small (Fig. 15F–J).

####### Distribution.

341–1800 m depth. New Zealand, Tasman Sea, Kermadec Islands, Bay of Plenty, Solomon Island, Coral Sea, Timor Sea, South Africa (off Glenmore), the South China Sea.

####### Remarks.

*Asteroschemasalix* was first described by Lyman (1879), then redescribed by Lyman (1882), Baker (1980), McKnight (2000), and Olbers et al. (2015). These redescriptions are useful to understand individual morphological character variation of *A.salix*. Specimens from our collection concur with previous redescriptions, but we noticed some variation such as: slightly separated granular ossicles on the disc, fewer granular ossicles on the ventral arm surface, and slightly longer arms. However, most of these morphological variations vary within individual specimens according to previous descriptions (Baker 1980; McKnight 2000). *Asteroschemasalix* strongly resembles *A.tubiferum*, *A.rubrum*, *A.laeve*, *A.inoratum*, *A.arenosum*, and *A.glaucum* but the characters of granulations and ossicle shape on the disc and arm, radial shield, and inner arm spine can be used to delimit *A.salix* from these species (Table 2). Previously, *A.salix* had been recorded from the South Pacific Ocean, and South African waters at a wide distribution range. This is the first record from the South China Sea.

###### ﻿ Asteroschema cf. lissum

Taxon classificationAnimaliaEuryalidaEuryalidae

H. L. Clark, 1939

07D71215-F3FD-5F08-9C88-ACF1CFA74132

Figures 16 , 17

Asteroschema lissum H. L. Clark, 1939: 37–39, figs 1–3.

####### Material examined.

China • 2 specimens; South China Sea, Zhongsha Islands, seamount; 13°36.20'N, 113°33.74'E; depth 1515 m; 30 Mar. 2020; Collecting event: stn. SC025; ‘Shenhaiyongshi’ msv leg; preserved in -80 °C; GenBank: OK044300; IDSSE-EEB-SW0079, IDSSE-EEB-SW080. Northwest Pacific • 1 specimen; near Mariana Trench, Southeast of Guam Island, seamount, 12°6.67'N, 141°37.27'E; depth 1160 m; 03 Sep. 2019; Collecting event: stn. SC033; ‘Shenhaiyongshi’ msv leg; preserved in -80 °C; GenBank: OK044299, OL712207; IDSSE-EEB-SW0081.

####### Description.

**Figure 16.**
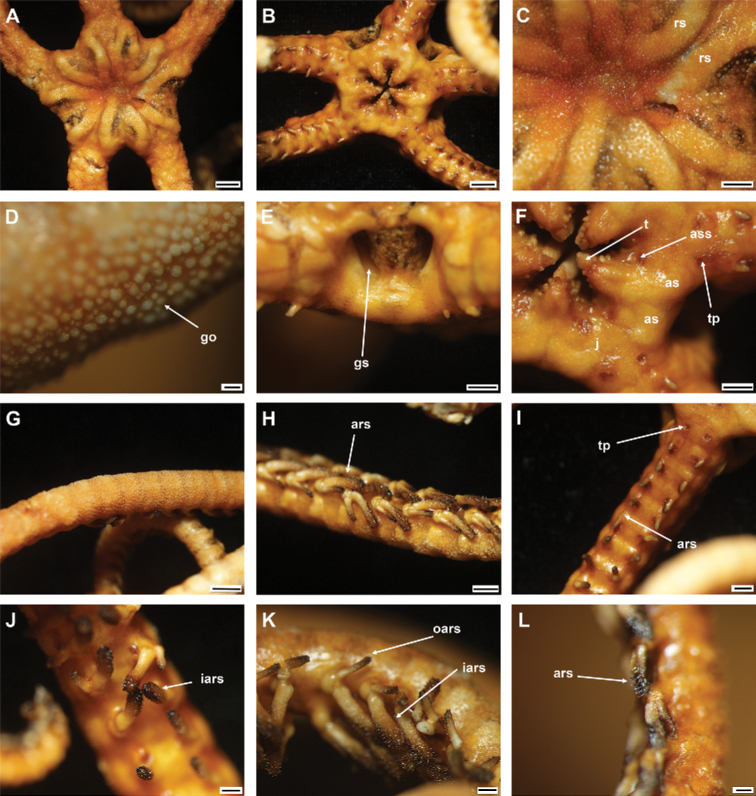
Asteroschemacf.lissum H. L. Clark, 1939 (IDSSE-EEB-SW0079) **A** dorsal view **B** ventral view **C** dorsal disc **D** skin on the disc **E** lateral disc **F** ventral disc **G** dorsal arm (middle) **H** lateral arm (middle) **I** ventral arm (proximal) **J** outer arm spine (proximal) **K** arm spines (middle) **L** arm spines (distal). Abbreviations: **as** adoral shield, **ass** adoral shield spine, **ars** arm spine, **go** granular ossicle, **gs** genital slit, **iars** inner arm spine, **j** jaw, **oars** outer arm spine, **ots** oral tentacle scale, **rs** radial shield, **t** teeth, **tp** tentacle pore. Scale bars: 2 mm (**A, B, G**); 1 mm (**C, E, F, H, I**); 500 µm (**J, K**); 200 µm (**D, L**).

IDSSE-EEB-SW0079: disc diameter 11.5 mm, length of arms 165 mm, arm base width 3–3.5 mm (Fig. 16).

***Disc*.** Disc flat, slightly raised above arms, swollen in center (Fig. 16A, B). Disc covered with smooth, small granular ossicles (Fig. 16C, D). Granular ossicles dense and small in disc center (six or eight grains in 1 mm), but slightly larger and separated at distal end of radial shield (five or six grains in 1 mm) (Fig. 16C). Radial shields wide, similar in size, curved, slightly raised above disc but not meeting in center (Fig. 16A, C). Radial shields clearly recognizable under thin skin embedded with ossicles (Fig. 16A, C). Genital slits narrow, concealed by polygonal granular ossicles, and vertical on ventral interradii (Fig. 16E). Jaws elongated, mostly naked without granular ossicles (Fig. 16F). Flattened, spearhead-shaped teeth, and six to seven granular ossicles that resemble lateral oral papillae at apex of jaw (Fig. 16F). Ventral disc covered with widely separated small granular ossicles (six or seven grains in 1 mm), but mostly covered by translucent thin skin (Fig. 16F). Adoral shields large, distal edge convex, concealed by thin skin with scattered small, smooth granular ossicles (Fig. 16F). Oral shields not discernible and oral tentacle pore naked without ossicles (Fig. 16F).

***Arms*.** Arms slightly arched, circular, from middle to distal half narrower and more cylindrical (Fig. 16G). Dorsal arm base covered with granular ossicles similar to dorsal disc (six or eight grains in 1 mm), increasingly separated and decreasing in size along the middle segments of the arm (seven or eight grains in 1 mm), distally widely separated (five or six grains in 1 mm) (Fig. 16G, H). On lateral arm plates, granular ossicles widely separated but continuing to base of arm (Fig. 16H, K). Only one or three ventral arm segments near arm base covered with few granular ossicles similar to ventral disc (six or seven grains in 1 mm), then completely naked along the arm (Fig. 16I, J). First arm spine appears at second arm segment, second arm spine at ninth or thirteenth segment (Fig. 16I–K). Inner arm spine initially short, thick with thorny pointed tip, at middle arm cylindrical, taller, one and a half arm segment in length, with flattened thorny tip (Fig. 16J, K). Outer arm spine half as long as inner spine in middle region, with smooth to thorny tip (Fig. 16K). Both arm spines equal in length at distal end of arm, and compound hook with five or six secondary teeth (Fig. 16L).

***Color*.** In live specimen, reddish brown but radial shields slightly lighter in color due to thin skin (Fig. 16).

####### Ossicle morphology.

**Figure 17.**
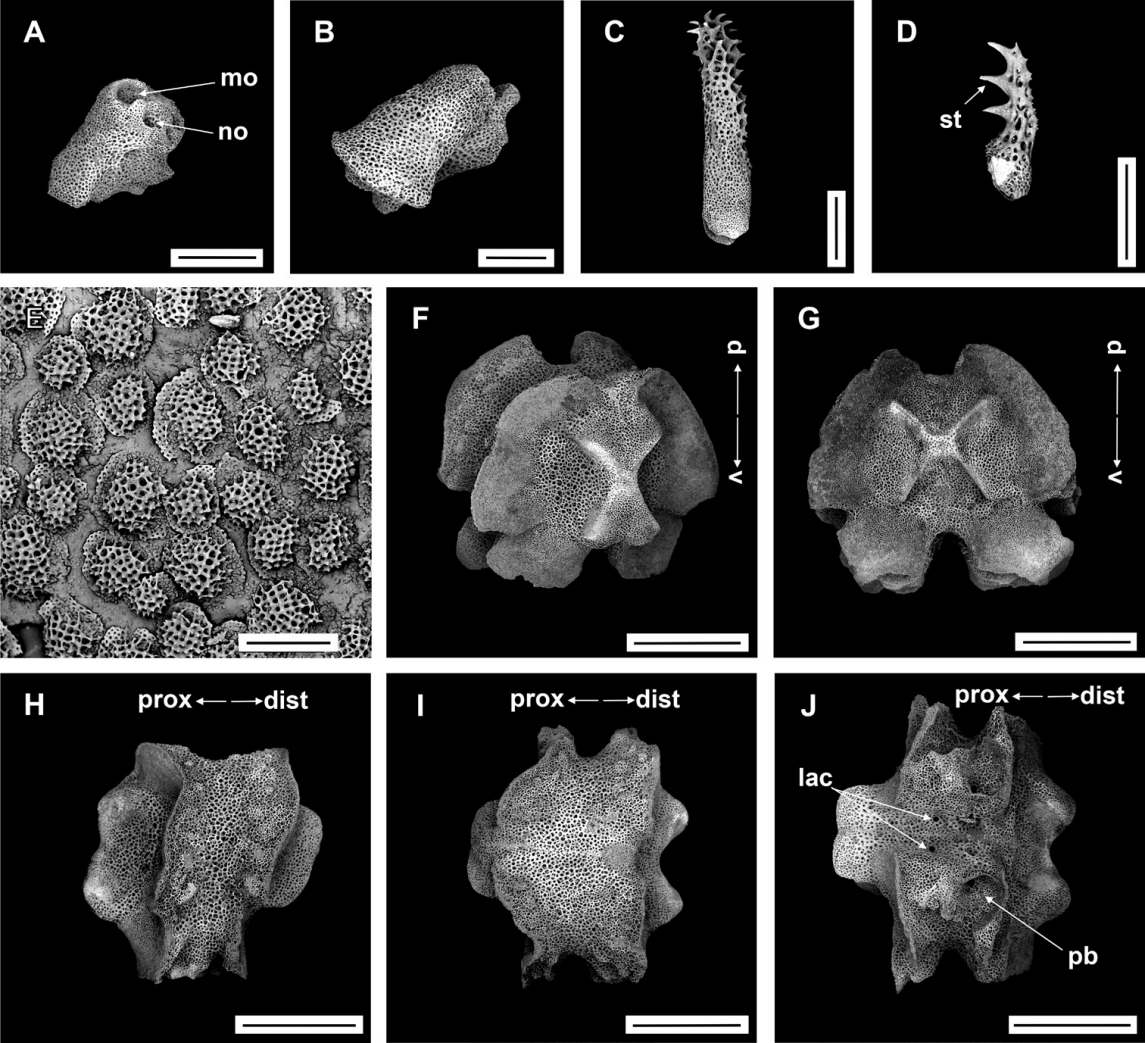
Asteroschemacf.lissum H. L. Clark, 1939 (IDSSE-EEB-SW0079) **A, B** lateral arm plate (external, internal) **C** outer arm spine (middle) **D** arm spine (distal) **E** skin from dorsal arm base (granular ossicles) **F–J** vertebrae **F** proximal view **G** distal view **H** lateral view **I** dorsal view **J** ventral view. Abbreviations: **d** dorsal, **dist** distal, **lac** lateral ambulacral canals, **mo** muscle opening, **no** nerve opening, **pb** podial basin, **prox** proximal, **st** secondary teeth, **v** ventral. Scale bars: 800 µm (**F–J**); 500 µm (**A, C**); 300 µm (**B, D**); 200 µm (**E**).

Lateral arm plate with two arm spine articular structures, with large muscle and nerve openings (Fig. 17A, B). Inner arm spine from proximal and middle half of arm cylindrical, with thorny tip (Fig. 17C). Distally, arm spine turns into compound hook with secondary teeth (Fig. 17D). Arm and disc concealed by less dense, wider, and short granular ossicles (Fig. 17E). Vertebrae with streptospondylous articulation, dorsally a median longitudinal furrow, ventrally with deep median longitudinal groove with lateral ambulacral canals, podial basins relatively small (Fig. 17F–J).

####### Distribution.

797–1515 m depth. Maldives, South China Sea, Northwest Pacific.

####### Remarks.

*Asteroschemalissum* was first described by H. L. Clark (1939), with type locality Maldives waters in the Indian Ocean. This is the first redescription after the original description. The specimens from our collection were close to H. L. Clark’s description but we noticed some differences, such as: characters of radial shields, and granular ossicles at ventral disc and base of arm. We hesitate to fully associate our specimens with *Asteroschemalissum* or propose a new species, due to uncertainty of these morphological variations. Some of these variations may be affected by size, maturity, and environment (holotype disc diameter 7.5 mm). *Asteroschemalissum* strongly resembles *A.hemigymnum*, *A.intectum*, and *A.sublaeve* by having similar granular density, and almost naked ventral disc and arms but differs in characters of the radial shields, start of second arm spine, granulation pattern on the disc and arm (Table 2). This is the first record of *A.lissum* from the South China Sea and the North Pacific Ocean, if it is indeed this species.

##### Genus *Asterostegus* Mortensen, 1933

###### ﻿ Asterostegus maini

Taxon classificationAnimaliaEuryalidaEuryalidae

McKnight, 2003

DE5CCD8C-5876-5558-A5FC-7F735C61DA7E

Figures 18 , 19

Asterostegus maini McKnight, 2003: 386–389, figs 1, 2.
Astroceras elegans McKnight, 1989: 25 (non Astroceraselegans Bell, 1917).

####### Material examined.

China • 2 specimens; South China Sea, near Xisha Islands archipelago, seamount; 16°47.79'N, 113°15.04'E; depth 602 m; 31 Mar. 2020; Collecting event: stn. SC009; ‘Shenhaiyongshi’ msv leg; preserved in -80 °C; GenBank: OK044303; IDSSE-EEB-SW0075; IDSSE-EEB-SW0076.

####### Description.

**Figure 18.**
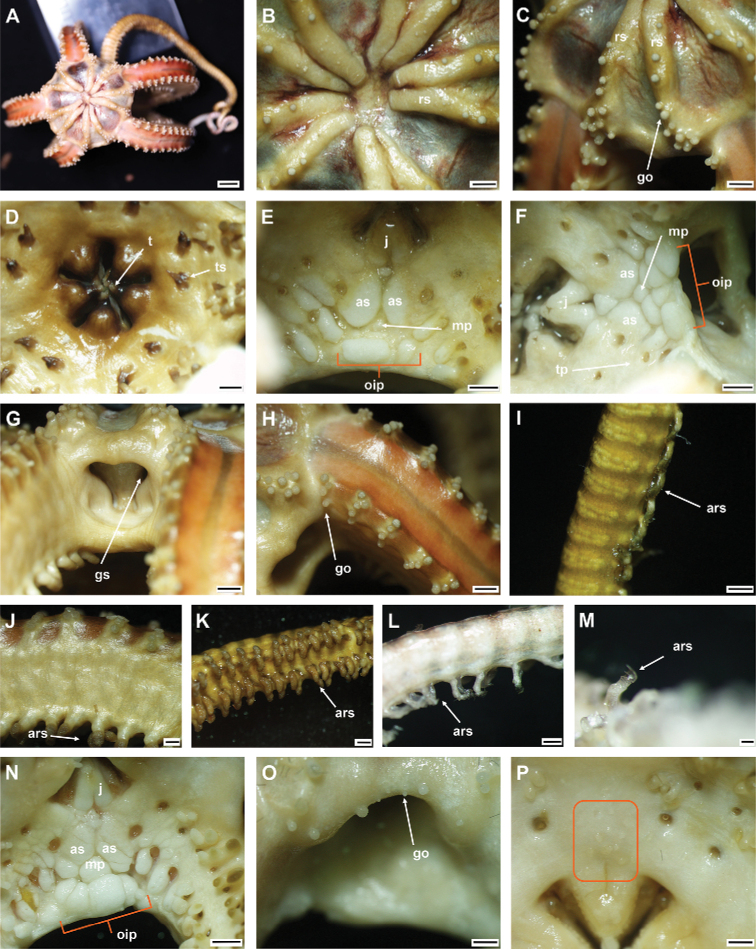
*Asterostegusmaini* McKnight, 2003 (**A–M**IDSSE-EEB-SW0076 **N–P**IDSSE-EEB-SW0075) **A** dorsal view **B** center of dorsal disc **C** dorsal disc (distal end of radial shields) **D** ventral disc **E, F** oral frame **G** lateral disc **H** dorsal arm (proximal) **I** dorsal arm (distal) **J** lateral arm (proximal) **K** ventral arm (middle) **L, M** arm spines (distal) **N** oral frame **O** periphery of the disc **P** oral frame (small transparent ossicles). Abbreviations: **as** adoral shield, **ars** arm spine, **go** granular ossicle, **gs** genital slit, **j** jaw, **mp** median plate, **oip** oral interradial plate, **rs** radial shield, **t** teeth, **tp** tentacle pore, **ts** tentacle scale. Scale bars: 6 mm (**A**); 2 mm (**B–H, N**); 1 mm (**I–K, O, P**); 500 µm (**L**); 200 µm (**M**).

IDSSE-EEB-SW0076: disc diameter 32.2 mm, length of arms 240–250 mm, height of arm base 9.8 mm (Fig. 18A–M).

***Disc*.** Disc circular and slightly inflated radially, with sunken interradial margins (Fig. 16A). Radial shields elongated, narrow, raised above the disc, extending nearly toward the disc center (Fig. 18B, C). Distal half of radial shield periphery covered by 10–12 (0.44 to 0.75 mm in width) large, club-shaped granules (Fig. 18C). Most of these granules cluster on distal end of radial shield (Fig. 18C). Entire disc, including radial shields, covered by thick skin (Fig. 18A–C). Adoral shields with slightly ovoid outline (Fig. 18E, F). Teeth spearhead-shaped, accompanied by granular domed lateral oral papillae but not visible in wet specimen (Fig. 18D–F). Proximally, adoral shields separated by triangular plate (Fig. 18F) Oral shields absent, and single or double row of two to six rounded, square, or irregular oral interradial plates beyond adoral shields (Fig. 18E, F). One median plate located between distal end of adoral shields, and slightly proximal to rest of oral interradial plates (Fig. 18E, F). Lateral interradial surface of disc slightly vertical and covered by thick naked skin, two conspicuous genital slits inside a large opening (Fig. 18G).

***Arms*.** Arms simple, strong, and not branching (Fig. 18A). Cross section of arm base slightly rectangular (7.2 mm in width and 8.5 mm high), but distal half of arm more cylindrical and narrower (Fig. 18H–J). Proximal to middle region of dorsal arm slightly flattened, ventral surface slightly arched, and lateral surface vertical (Fig. 18J, K). Whole arm concealed by thick skin (Fig. 18H–K). Lateral arm plate on proximal half of arms bears three to five club-shaped granules (0.4–0.6 mm in width) (Fig. 18H), decreasing in size and number along arm and vanishing on distal half of arm (Fig. 18I). Ventral arm covered by naked skin (Fig. 18K). First one to two tentacle pores lack arm spines; second or third pore with two or three arm spines, similar in size (Fig. 18D). At proximal end of arms, arm spines short, thick, ovoid with more or less rounded tip with rough surface, at middle of arms club-shaped, transversely flattened (Fig. 18J, K). Arm spines turn into hook with two to three secondary teeth at distal end of arms (Fig. 18L, M).

***Color*.** In live specimen, dorsal disc interradially dark brown but radial shields light brown. Ventral and lateral disc whitish brown, dorsal surface of proximal and middle regions of arms bright brown/red. Lateral and ventral surface of arms whitish brown, granules creamy white (Fig. 18).

####### Ossicle morphology.

**Figure 19.**
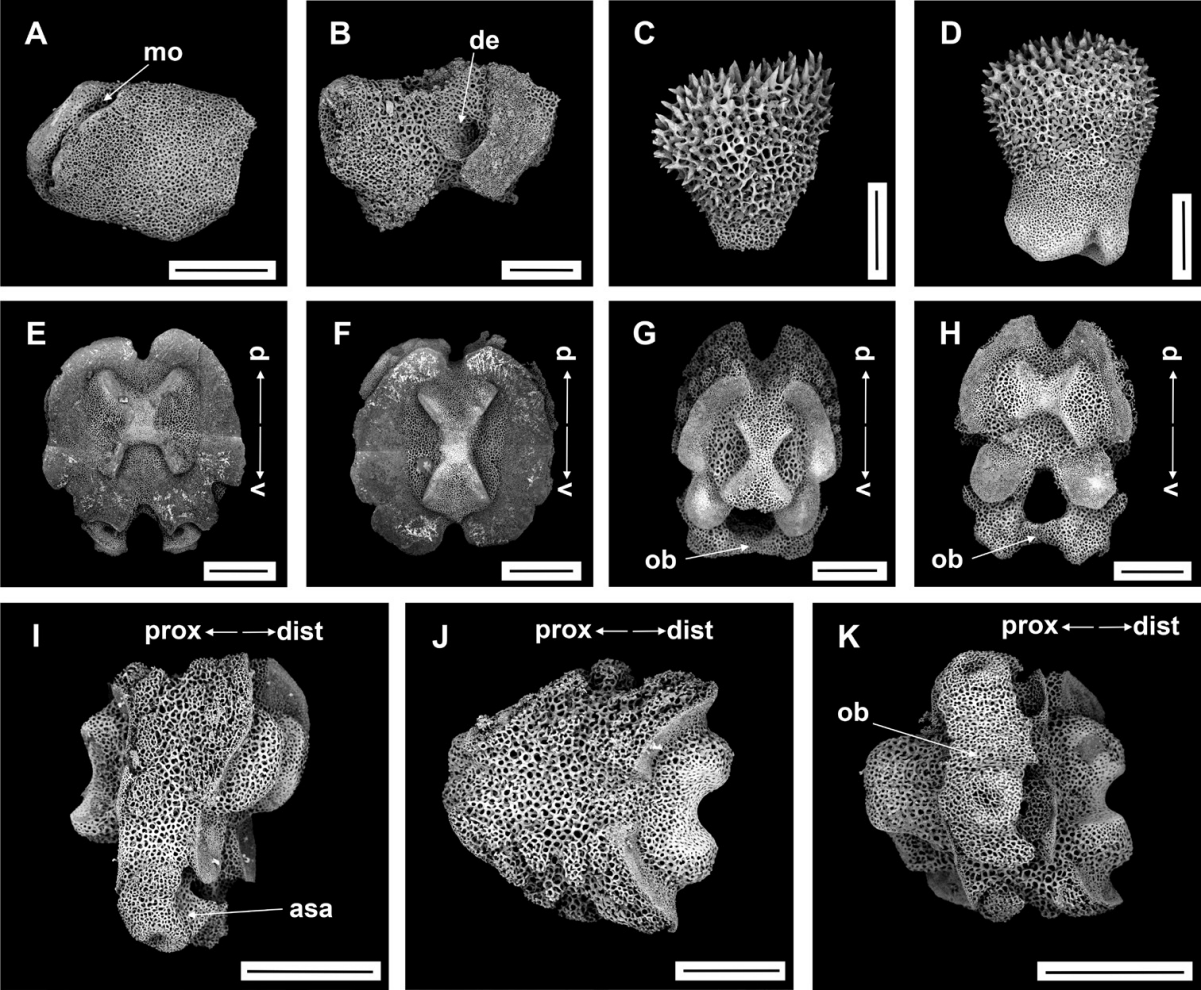
*Asterostegusmaini* McKnight, 2003 (IDSSE-EEB-SW0076) **A, B** lateral arm plate (external, internal) **C, D** arm spine (middle) **E, F** vertebrae (middle) **E** proximal view **F** distal view **G–K** vertebrae (distal) **G** proximal view **H** distal view **I** lateral view **J** dorsal view **K** ventral view. Abbreviations: **asa** arm spine articular structure, **d** dorsal, **de** depression, **dist** distal, **mo** muscle opening, **ob** oral bridge, **prox** proximal, **v** ventral. Scale bars: 800 µm (**E, F**); 500 µm (**A, C, D, I, K**); 300 µm (**B, G, H, J**).

Lateral arm plate with two arm spine articular structures, middle half of arm with large and wide, separated muscle and nerve openings, depression on inner side (Fig. 19A, B). Arm spines large, short, flattened, and club-shaped with thorny surface (Fig. 19C, D). Vertebrae with streptospondylous articulation. Vertebrae on proximal to middle half of the arm large, flat with deep ventral groove, no oral bridge (Fig. 19E, F). Vertebrae on distal half of arm slightly longer, dorsally large median longitudinal furrow, and deep median longitudinal groove on ventral side, with oral bridge (Fig. 19G, H).

####### Distribution.

417–602 m in depth. New Zealand (Cook Island), South China Sea.

####### Remarks.

*Asterostegusmaini* was first described by McKnight (2003), with type locality Cook Islands, South Pacific Ocean, and Okanishi and Fujita (2014) redescribed it. However, this is the first record of *Asterostegusmaini* since the holotype. Here we recorded two specimens from the South China Sea (disc diameter 26 mm and 32.2 mm) and both are larger than the holotype (disc diameter 22 mm). Currently, three species belong to the genus *Asterostegus*: *A.maini* McKnight, 2003, *A.tuberculatus* Mortensen, 1933, and *A.sabineae* Okanishi & Fujita, 2014. *Asterostegustuberculatus* differs from *A.maini* in granules being scattered across the whole disc including the radial shield, and only two to three stump-like granules on the dorsolateral arm plate (McKnight 2003; Okanishi and Fujita 2014). *Asterostegussabineae* differs from *A.maini* in large stump-like granules on the radial shield, only one oral interradial plate, and one or two large stump-like granules on the dorsolateral arm plate (Okanishi and Fujita 2014). The number of oral interradial plates and their arrangement are highly variable within and between individuals (Fig. 18E, F, N). Previous studies on the holotype showed only one row with two to five interradial plates (McKnight 2003; Okanishi and Fujita 2014). However, in our specimens, these are arranged in one or two rows with two to seven interradial plates in total (Fig. 18E, F, N). Therefore, one of the key morphological characters in the genus *Asterostegus*, the arrangement of oral interradial plates has to be modified. One specimen from the present study (IDSSE-EEB-SW0075, 26 mm disc diameter) showed some morphological variations, such as: small granules on the periphery of the disc and on few small areas on the ventral disc (Fig. 18O, P). These granules are extremely small compared to those on the radial shields and arms. In the specimens from the present study, first arm spines started from second or third arm segment, but in the holotype, it started from the fourth arm segment. However, except for these small morphological variations, both specimens were similar to the holotype description.

#### Family Gorgonocephalidae Ljungman, 1867

**Subfamily Gorgonocephalinae Döderlein, 1911**

##### Genus *Astrodendrum* Döderlein, 1911

###### ﻿ Astrodendrum cf. sagaminum

Taxon classificationAnimaliaEuryalidaGorgonocephalidae

(Döderlein, 1902)

0F632D74-B933-5921-9C03-B674FFFE18FD

Figures 20 , 21

Astrodendrum sagaminum Döderlein, 1902: 321–322; 1911: 38–39, figs 2, 3–5, 7, 8; 1927: 32, 92; H.L. Clark 1911: 292–293; A. H. Clark 1916a: 185; Liao 2004: 109–111, fig. 52.

####### Material examined.

China • 1 specimen; South China Sea, East side from Zhongsha Islands, seamount; 16°22.11'N, 116°06.60'E; depth 1619 m; 09 Aug. 2020; Collecting event: stn. SC028; ‘Shenhaiyongshi’ msv leg; preserved in -80 °C; GenBank: OK044304; IDSSE-EEB-SW0104.

####### Description.

**Figure 20.**
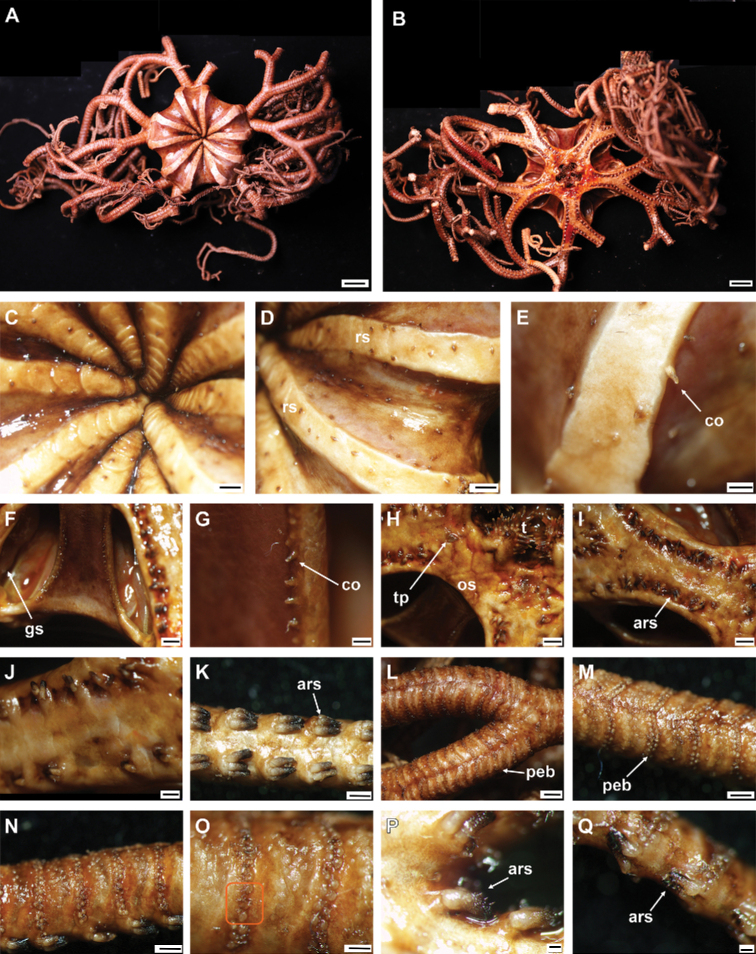
Astrodendrumcf.sagaminum (Döderlein, 1902) (IDSSE-EEB-SW0104) **A** dorsal view **B** ventral view **C** dorsal disc (center) **D** dorsal disc (distal edge) **E** radial shield **F, G** ventral disc **H** oral frame **I, J** ventral view of arm base **K** ventral view of after second arm branch **L, M** dorsal view of second arm branch **N** lateral view of arm (middle) **O** lateral view of arm (pedicellariae with baseplate) **P, Q** variations of arm spine on distal end of the arm. Abbreviations: **ars** arm spine, **co** conical ossicles **gs** genital slit, **os** oral shield, **peb** = pedicellarial band, **rs** radial shield, **t** teeth, **tp** tentacle pore. Scale bars: 16 mm (**A, B**); 2 mm (**C, D, F, L**); 1 mm (**E, H–K, M, N**); 500 µm (**G, O**); 200 µm (**P, Q**).

Disc diameter 62 mm (Fig. 20A, B).

***Disc*.** Dorsal disc slightly inflated, swollen in the center (Fig. 20A, B). Radial shields elongated, tapered at proximal end, extending to center of disc (Fig. 20C, D). Entire disc covered by skin with conical ossicles of various size (0.4–0.7 mm high) and widely separated and scattered (Fig. 20C–E). Genital slits conspicuous, interradial margin covered by two rows of higher than wide conical ossicles (Fig. 20F, G). Ventral disc almost naked, but micro-granular ossicles visible on oral region (Fig. 18H). Oral area covered by smooth skin with few scattered small granular ossicles, exposing adoral and oral shield outlines (Fig. 20H). Oral plates flat, polygonal, and slightly in contact with adoral shields (Fig. 20H). Adoral shields short, square. Oral papillae and teeth spiniform (Fig. 20H), several vertical rows of teeth on dental plate (possibly tooth papillae at ventral edge).

***Arms*.** Arms branched at least eight to nine times, flexible dorso-ventrally, flat ventrally, arched dorsally (Fig. 20I–N). Ventral arm surface covered by smooth skin; proximal half with widely scattered small, flat, polygonal granular ossicles (Fig. 20I–K). Dorsal arm surface covered by polygonal or domed plates and between these pedicellarial bands (Fig. 20L–O) that appear after second arm fork, covering whole lateral to dorsal area of arm, creating annulated appearance (Fig. 20L). First arm segment lacks spines, next four to six with two arm spines, thereafter two or three arm spines per segment (Fig. 20I–K). Ventral arm spines similar in size, smaller, unevenly pointed, distally turning into hooks with 2–3 secondary teeth (Fig. 20K, P, Q).

***Color*.** In live specimen, whole specimen brown, but radial shields, oral regions, and ventral arms lighter than disc (Fig. 20).

####### Ossicle morphology.

**Figure 21.**
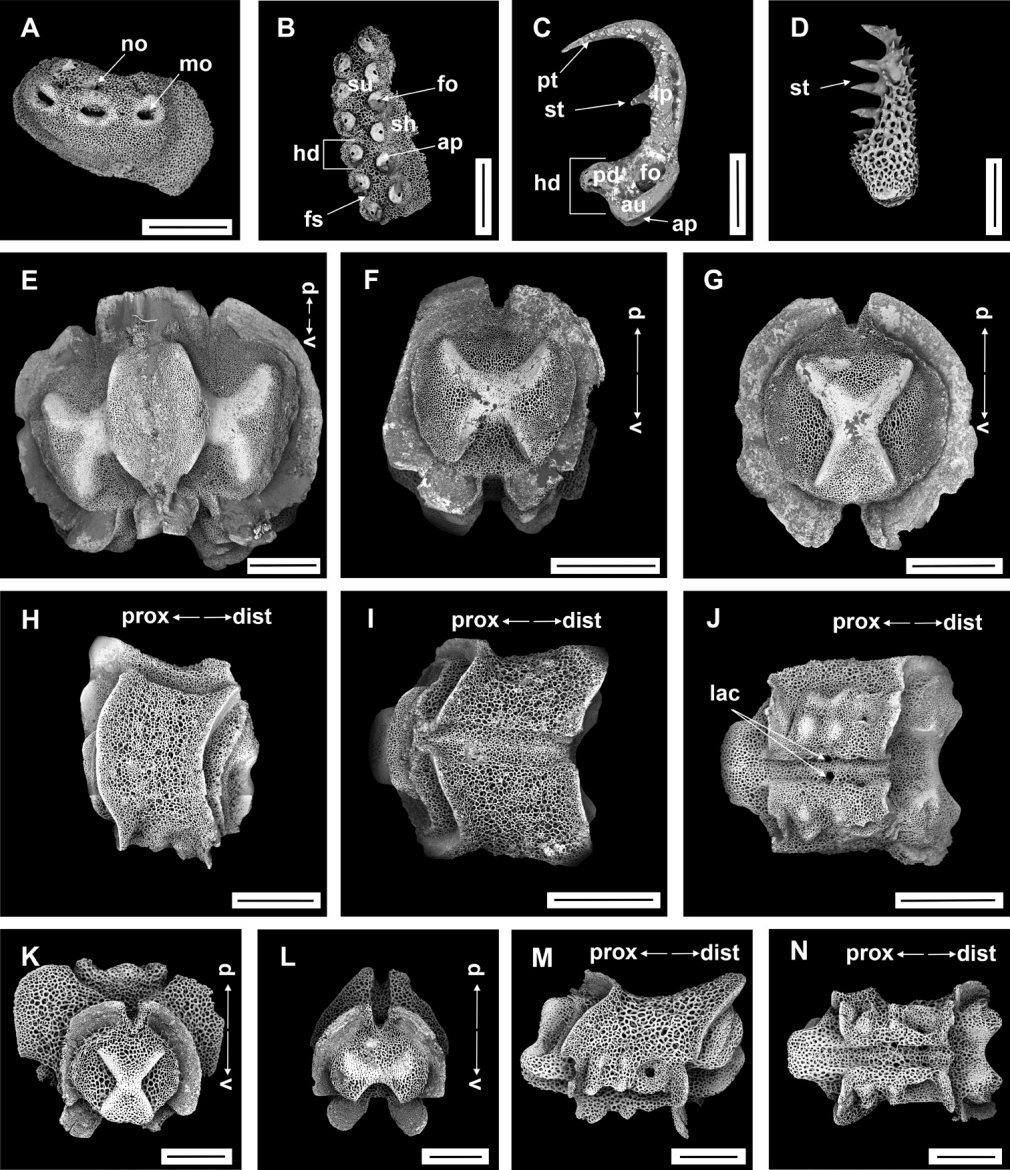
Astrodendrumcf.sagaminum (Döderlein, 1902) (IDSSE-EEB-SW0104) **A** lateral arm plate (middle) **B** plan view of baseplate **C** pedicellariae **D** arm spine on distal end **E–J** vertebrae on middle half of arm **E** proximal view of branch vertebrae **F** proximal view **G** distal view **H** lateral view **I** dorsal view **J** ventral view **K–N** vertebrae on distal end of arm **K** distal view **L** proximal view **M** lateral view **N** ventral view. Abbreviations: **ap** articular pad of the base, **au** auricle, **d** dorsal, **dist** distal, **fo** foramina of the base, **fs** fossa between adjacent tubercles, **hd** head of the apophysis, **lac** lateral ambulacral canals, **mo** muscle opening, **no** nerve opening, **pd** pedicel of the apophysis, **prox** proximal, **pt** primary tooth of the blade, **sh** sheath of the baseplate, **su** sulcus of tubercle head, **v** ventral. Scale bars: 800 µm (**E–J**); 500 µm (**A, B**); 300 µm (**K–N**); 200 µm (**D**); 80 µm (**C**).

On middle half of arm, lateral arm plates with perforations on ventral side, large muscle opening and small nerve opening (Fig. 21A). Pedicellarial bands formed by approximately 12 articulating tubercles at curved distal end of baseplate and these articulations have a single foramen per tubercle for pedicellariae with one secondary tooth (Fig. 21B, C). Ventral arm spines on distal end of arm transformed into hook with two or three secondary teeth (Fig. 21D). Pedicellariae differ from ventral arm spine by having smooth apophysis (Fig. 21C, D). Vertebrae with streptospondylous articulation with smooth lateral furrows and paired openings in lateral side of vertebrae for lateral ambulacral canals, no oral bridge (Fig. 21F–N).

####### Distribution.

90–1300 m depth. South China Sea, Japan, East China Sea, Sri Lanka.

####### Remarks.

The specimen is similar to the holotype description by Döderlein (1902), and the redescriptions of Döderlein (1911, 1927), Liao (2004) and Okanishi and Fujita (2018), but showed some morphological variations especially on the disc (Fig. 20). Therefore, we hesitate to fully associate our specimen with *Astrodendrumsagaminum*. All descriptions mentioned granules on both dorsal and ventral disc, but in the present specimen, the dorsal disc is covered with widely separated conical stump-like granules and the ventral disc is covered with widely scattered micro-polygonal ossicle plates in naked skin. However, Baker (1980) mentioned that the granular pattern on the disc was not a suitable morphological feature to delimit species in the genus *Gorgonocephalus*. This is the first record of *Astrodendrumsagaminum* from the South China Sea, if this is indeed that species.

## ﻿Discussion

The molecular phylogenetic trees of these species of Gorgonocephalidae and Euryalidae were in agreement with previous studies (Okanishi and Fujita 2013; Christodoulou et al. 2019; O’Hara et al. 2019). Previous molecular studies indicated that intraspecific genetic distance ranges approximately from 0.5% to 6.4%, with a mean of 2.2%, but species from the family Euryalidae usually showed less than 2% mean genetic distance (Okanishi et al. 2011, 2018; Okanishi and Fujita 2013; Boissin et al. 2017). In this study, we focused on the genera *Asteroschema*, *Asterostegus*, and *Astrodendrum*. In addition, we included species from the genus *Ophiocreas* due to their similar morphology to *Asteroschema* species. The species in the genus *Asteroschema* were difficult to analyze only morphologically due to great similarity in most morphological characters. Previous studies divided *Asteroschema* into three groups according to ossicle shape on the disc and arms, but still many species within these groups are hard to identify. In this study, we successfully managed to obtain the COI and 16S sequences from only one of the proposed new species, *Asteroschemashenhaiyongshii* sp. nov., which suggests a close relationship to *A.bidwillae* according to genetic distance (2.56%), but *A.shenhaiyongshii* sp. nov. is pentamerous and shows no signs of fission. It also has dense granular ossicle coverage on the ventral disc and ventral arm surface (Suppl. material 2: Table S2; Fig. 6). Therefore, we consider these two as sibling species. According to the present study, we suggest a species complex within *Asteroschematubiferum* due to morphological variations between specimens from the South China Sea and New Zealand. However, we found significantly low genetic distances between the specimens identified as A.cf.lissum, *A.tubiferum*, *A.rubrum*, and *A.salix* (2.79±0.66% SE) (Suppl. material 2: Table S2). Therefore, understanding key morphological differences and intraspecific genetic distance range are important to delimit *Asteroschema* species. Asteroschemacf.lissum was recognized here as intermediate species between *A.salix* and *A.tubiferum* due to genetic distance values between these species. In the ML tree of the family Euryalidae, all *Ophiocreas* species cluster with *Asteroschemaoligactes*, *A.migrator*, *A.edmondsoni*, *A.ajax*, and *A.horridum.* All these *Asteroschema* species have conical ossicles or annular bands on the arms. These two clades may correspond to one of these genera each or one of them may belong to both genera (making them synonymous) and the other to a putative new genus, but since the type species of both genera have not been sequenced yet, it is impossible to decide. Thus, the present study concurs with previous molecular studies in the hypothesis that *Asteroschema* may be polyphyletic, but may instead be paraphyletic with the genus *Ophiocreas*, and the present morphological differentiation between these two genera can be questioned (Okanishi and Fujita 2013; Christodoulou et al. 2019; O’Hara et al. 2019). A comprehensive morphological and molecular taxonomic revision, including examination of type specimens of all *Asteroschema* and *Ophiocreas* species is needed to understand the key morphological characters and genetic differences. We tentatively place our new species in *Asteroschema*, but acknowledge that they may later be found to belong in *Ophiocreas*.

The genus *Asterostegus* includes only three species, and is closely related to *Astroceras*, but a previous phylogenetic analysis recognized it as monophyletic and belonging in the family Euryalidae (Okanishi and Fujita 2013). The interspecific genetic distance (3.09±0.75% SE) within the genus *Asterostegus* was low and similar to other Euryalidae species (Suppl. material 2: Table S2). The genetic distance of the genus *Gorgonocephalus* was significantly lower than in *Asteroschema*. The molecular phylogenetic analysis of *Astrodendrum* and *Gorgonocephalus* showed two main clades, and previous studies showed that *Gorgonocephalus* may be polyphyletic (Okanishi and Fujita 2013; Christodoulou et al. 2019; O’Hara et al. 2019). In the present study, Astrodendrumcf.sagaminum clustered with *Gorgonocephalussundanus* and *G.pustulatum*. The type species *Gorgonocephaluscaputmedusae* (Linnaeus, 1758) clustered with *G.chilensis*, *G.eucnemis*, *G.arcticus*, and *G.tuberosus* (Fig. 3, Suppl. material 3: Table S3). We suggest that *A.sagaminum* should belong in the genus *Gorgonocephalus*, or alternatively, *Gorgonocephalus* could be split into two genera, but a more thorough study with more genes and more specimens should be performed, before this step is taken.

Most of the species from the present study were collected from deep water in the South China Sea. Previous studies from the South China Sea recorded only few *Asteroschema* species, but found no representatives of the genera *Astrodendrum* and *Asterostegus*. According to the present study, the ophiuroid diversity of the South China Sea may be higher than previously known and future expeditions to the South China Sea deep-sea seamounts may discover even more species. The present study suggests a wider distribution of Euryalida species from the South-Pacific to the North-Pacific regions than previously expected.

## Supplementary Material

XML Treatment for Asteroschema domogranulatum

XML Treatment for Asteroschema shenhaiyongshii

XML Treatment for Asteroschema cf. bidwillae

XML Treatment for Asteroschema rubrum

XML Treatment for Asteroschema tubiferum

XML Treatment for Asteroschema salix

XML Treatment for Asteroschema cf. lissum

XML Treatment for Asterostegus maini

XML Treatment for Astrodendrum cf. sagaminum

## Supplementary materials

Supplementary material 1 Table S1 Data type Primers (text) Explanation note COI and 16S primers and PCR cycles used in this study (Hoareau and Boissin 2010; Okanishi and Fujita 2013). https://binary.pensoft.net/file/665898This dataset is made available under the Open Database License (http://opendatacommons.org/licenses/odbl/1.0/). The Open Database License (ODbL) is a license agreement intended to allow users to freely share, modify, and use this Dataset while maintaining this same freedom for others, provided that the original source and author(s) are credited.Hasitha Nethupul, Sabine Stöhr, Haibin Zhang

Supplementary material 2 Table S2 Data type Genetic distance % (COI) Explanation note Genera *Asteroschema*, *Ophiocreas* and *Asterostegus*, pairwise distance values based on 592 bp mitochondrial COI sequences, calculated using the Kimura 2-parameter method with 1,000 bootstrap replicates (Value in blue color represent Standard Error). Abbreviations: AUS Australia, CAN Canada, CS Caribbean Sea, JAP Japan, MAD Madagascar, NCA New Caledonia, NWP Northwest Pacific, NZ New Zealand, PAO Pacific Ocean, SCS South China Sea. https://binary.pensoft.net/file/665899This dataset is made available under the Open Database License (http://opendatacommons.org/licenses/odbl/1.0/). The Open Database License (ODbL) is a license agreement intended to allow users to freely share, modify, and use this Dataset while maintaining this same freedom for others, provided that the original source and author(s) are credited.Hasitha Nethupul, Sabine Stöhr, Haibin Zhang

Supplementary material 3 Table S3 Data type Genetic distance % (COI) Explanation note Genera *Gorgonocephalus* and *Astrodendrum*, pairwise distance values based on 730 bp mitochondrial COI sequences, calculated using the Kimura 2-parameter method with 1,000 bootstrap replicates (Value in blue color represent Standard Error). Abbreviations: AN Antarctic Sea, AUS Australia, CAN Canada, JAP Japan, NZ New Zealand, SCS South China Sea, SWE Sweden. https://binary.pensoft.net/file/665900This dataset is made available under the Open Database License (http://opendatacommons.org/licenses/odbl/1.0/). The Open Database License (ODbL) is a license agreement intended to allow users to freely share, modify, and use this Dataset while maintaining this same freedom for others, provided that the original source and author(s) are credited.Hasitha Nethupul, Sabine Stöhr, Haibin Zhang

## References

[B1] Alcock A (1894) Natural history notes from H.M. Indian Marine Survey Steamer Investigator, Commander C.F. Oldham, R.N., commanding. Series 2, No 9. An account of the deep-sea collection made during the season of 1892–93. Journal of the Asiatic Society of Bengal 62: 169–184. https://www.biodiversitylibrary.org/page/37190562.

[B2] Baker AN (1980) Euryalinid Ophiuroidea (Echinodermata) from Australia, New Zealand, and the south-west Pacific Ocean. New Zealand Journal of Zoology 7: 11–83. 10.1080/03014223.1980.10423763

[B3] Boissin E Hoareau TB Paulay G Bruggemann JH (2017) DNA barcoding of reef brittle stars (Ophiuroidea, Echinodermata) from the southwestern Indian Ocean evolutionary hot spot of biodiversity. Ecology and Evolution 7: 11197–11203. 10.1002/ece3.3554 29299292 PMC5743570

[B4] Byrne M (1994) Ophiuroidea. In: Echinodermata. Microscopic Anatomy of Invertebrates. Wiley-Liss, New York, 247–343.

[B5] Christodoulou M O’Hara TD Hugall AF Arbizu PM (2019) Dark Ophiuroid Biodiversity in a Prospective Abyssal Mine Field. Current Biology 29: 3909–3912. 10.1016/j.cub.2019.09.012 31630951

[B6] Clark AH (1916a) XV. One new starfish and five new brittle stars from the Galápagos Islands. Annals and Magazine of Natural History 18: 115–122. 10.1080/00222931608693831

[B7] Clark AH (1949) Ophiuroidea of the Hawaiian Islands. Bulletin of the Bernice P. Bishop Museum 195: 3–133.

[B8] Clark HL (1911) North Pacific Ophiurans in the collection of the United States National Museum. Smithsonian Institution United States National Museum Bulletin 75: 1–302. 10.5479/si.03629236.75.1

[B9] Clark HL (1915) Catalogue of recent ophiurans, based on the collection of the Museum of Comparative Zoology. Memoirs of the Museum of comparative Zoology at Harvard College 25: 164–376. 10.5962/bhl.title.48598

[B10] Clark HL (1916b) Report on the sea-lilies, starfishes, brittle-stars and sea-urchins obtained by the F.I.S. “Endeavour” on the coasts of Queensland, New South Wales, Tasmania, Victoria, South Australia, and Western Australia. Biological Results of the Fishing experiments carried on by the F.I.S. Endeavour 1909–1914 4: 1–123. 10.5962/bhl.title.13854

[B11] Clark HL (1917) Reports on the Scientific Results of the Albatross Expedition to the Tropical Pacific, 1899–1900 (Part 18). Reports on the Scientific results of the Albatross Expedition to the Eastern Tropical Pacific, 1904–1905 (Part 30). Ophiuroidea. Bulletin of the Museum of Comparative Zoology at Harvard 61: 429–453.

[B12] Clark HL (1939) Ophiuroidea. Scientific Reports from the John Murray Exp. 1933–34 6: 29–136.

[B13] Clark HL (1941) Reports on the scientific results of the Atlantis expeditions to the West Indies, under the joint auspices of the University of Havana and Harvard University. The Echinoderms (other than holothurians). Memorias de la Sociedad Cubana de Historia Natural 15: 1–154.

[B14] Döderlein L (1911) Über japanische und andere Euryalae. Abhandlungen der math. phys. Klasse der K. Bayerischen Akademie der Wissenschaften, Suppl 5: 1–123. https://www.biodiversitylibrary.org/page/16339443

[B15] Döderlein L (1927) Indopacifische Euryalae. Abhandlungen der Bayerischen Akademie der wissenschaften 31: 1–106. 10.1515/9783486755459

[B16] Döderlein L (1930) Die Ophiuroiden der Deutschen Tiefsee-Expedition. 2. Euryale. Wissenschaftliche Ergebnisse der Deutschen Tiefsee-Expedition auf dem Dampfer “Valdivia” 1898–1899. 22.

[B17] FWRI (2010) FWC Fish and Wildlife Research Institute’s albums. https://www.flickr.com/photos/myfwc/15147472218/in/album-72157639735390743/ [accessed 5 December 2021]

[B18] Goharimanesh M Stöhr S Mirshamsi O Ghassemzadeh F Adriaens D (2021) Interactive identification key to all brittle star families (Echinodermata; Ophiuroidea) leads to revised morphological descriptions. European Journal of Taxonomy 766: 1–63. 10.5852/ejt.2021.766.1483

[B19] Guille A (1981) 91 Mémoires du Muséum national d’Histoire naturelle Echinodermes: Ophiurides. du Muséum nationain: Forest J (Ed.) Résultats des campagnes MUSORSTOM: 1. Philippines (18–28 Mars 1976). Résultats, 413–456 pp.

[B20] Hendler G (2018) Armed to the teeth: A new paradigm for the buccal skeleton of brittle stars (Echinodermata: Ophiuroidea). Contributions in Science 526: 189–311. 10.5962/p.324539

[B21] Hoareau TB Boissin E (2010) Design of phylum-specific hybrid primers for DNA barcoding: Addressing the need for efficient COI amplification in the Echinodermata. Molecular Ecology Resources 10: 960–967. 10.1111/j.1755-0998.2010.02848.x 21565105

[B22] International Hydrographic Organization [IHO], Sieger R (2012) Names of oceans and seas as digitized table. Alfred Wegener Institute, Helmholtz Centre for Polar and Marine Research, Bremerhaven. 10.1594/PANGAEA.777976

[B23] Kimura M (1980) A simple method for estimating evolutionary rates of base substitutions through comparative studies of nucleotide sequences. Journal of Molecular Evolution 16: 111–120. 10.1007/BF01731581 7463489

[B24] Koehler R (1904) Ophiures de l’expédition du Siboga. Part 1. Ophiures de mer profonde. Weber, Siboga Expeditie. MEJ Brill, Leiden 45a: 1–176. 10.5962/bhl.title.11682

[B25] Koehler R (1906) Description des Ophiures nouvelles recueilles par le Travailleur et Talisman pendant les campagnes de 1880, 1881, 1882 et 1883. Mémoires de la Société zoologique de France 19: 5–35.

[B26] Koehler R (1907) 41 Révision de la collection des ophiures du Muséum d’Histoire naturelle de Paris. Bulletin scientifique de la France et de la Belgique, 279–351.

[B27] Koehler R (1914) A contribution to the study of Ophiurans of the United States National Museum. Bulletin of the United States National Museum 84: 1–173. https://biodiversitylibrary.org/page/7907416

[B28] Koehler R (1930) Ophiures recueillies par le Docteur Th. Mortensen dans les Mers d’Australie et dans l’Archipel Malais. Papers from Dr. Th. Mortensen’s Pacific Expedition 1914–16. LIV. Videnskabelige Meddelelser fra Dansk naturhistorisk Forening 89: 1–295.

[B29] Kumar S Stecher G Tamura K (2016) MEGA7: Molecular Evolutionary Genetics Analysis Version 7.0 for Bigger Datasets. Molecular Biology and Evolution 33: 1870–1874. 10.1093/molbev/msw054 27004904 PMC8210823

[B30] Kumar S Stecher G Li M Knyaz C Tamura K (2018) MEGA X: Molecular evolutionary genetics analysis across computing platforms. Molecular Biology and Evolution 35: 1547–1549. 10.1093/molbev/msy096 29722887 PMC5967553

[B31] Lamarck J-B de (1816) Ordre Second. Radiaires Échinodermes. Histoire naturelle des animaux sans vertèbres 2: 522–568. https://www.biodiversitylibrary.org/page/13299296

[B32] Leach WE (1819) Descriptions des nouvelles espèces d’Animaux découvertes par le vaisseau ‘Isabelle’ dans un voyage au pôle boréal. Journal de Physique, de Chimie, et d’Histoire Naturelle 88: 462–467. http://www.biodiversitylibrary.org/item/29607#

[B33] Liao Y (2004) Echinodermata: Ophiuroidea. Fauna Sinica: Zoology of China Invertebrates 40: 1–305. [pls I–VI]

[B34] Linnaeus C (1758) Systema Naturae per regna tria naturae, secundum classes, ordines, genera, species, cum characteribus, differentiis, synonymis, locis. Editio decima, reformata [10th revised edition], Laurentius Salvius, Holmiae, vol. 1, 824 pp. 10.5962/bhl.title.542

[B35] Ljungman A (1867) Ophiuroidea viventia huc usque cognita enumerat. Öfversigt af Kgl. Vetenskaps-Akademiens Förhandlingar 1866 23: 303–336. https://www.biodiversitylibrary.org/page/32287761

[B36] Ljungman AV (1872) Förteckning öfver uti Vestindien af Dr A. Goës samt under korvetten Josefinas expedition i Atlantiska Oceanen samlade Ophiurider. Öfversigt af Kungliga Vetenskapsakademiens Förhandlingar 28: 615–658.

[B37] Lütken CF Mortensen T (1889) Reports on an exploration off the west coasts of Mexico, Central and Southern America and off the Galapagos Islands. XXV. The Ophiuridae. Memoirs of the Museum of Comparative Zoology 23: 97–208. https://www.biodiversitylibrary.org/page/28891692

[B38] Lyman T (1869) Preliminary report on the Ophiuridae and Astrophytidae dredged in deep water between Cuba and Florida Reef. Bulletin of the Museum of Comparative Zoology 1: 309–354. https://biodiversitylibrary.org/page/6587804.

[B39] Lyman T (1872) Note sur les Ophiurides et Euryales qui se trouvent les collections de Muséum d’Histoire Naturelle de Paris. Annales des Sciences Naturelles, series 5, Zoologie 16: 3–8. https://www.biodiversitylibrary.org/page/33076719.

[B40] Lyman T (1875) Zoological Results of the Hassler Expedition. 2. Ophiuridae and Astrophytidae. Illustrated catalogue of the Museum of Comparative Zoology at Harvard College 8: 1–34.

[B41] Lyman T (1878) Ophiurans and Astrophytons. Reports on the dredging operations of the US coast survey Str. “Blake.” Bulletin of the Museum of Comparative Zoology 5: 217–238. https://www.biodiversitylibrary.org/page/30295874

[B42] Lyman T (1879) Ophiuridae and Astrophytidae of the “Challenger” expedition. Part II. Bulletin of the Museum of Comparative Zoology at Harvard College, Cambridge, Mass. 6: 17–83. https://www.biodiversitylibrary.org/page/31068674#page/27/mode/1up

[B43] Lyman T (1882) Ophiuroidea. Bulletin of the Museum of Comparative Zoology at Harvard College, Cambridge, Mass. Scientific Reports. Results voy. H.M.S. “Challenger”, 388 pp.

[B44] Lyman T (1883) Reports on the results of dredging, under the supervision of Alexander Agassiz, in the Caribbean Sea (1878–79), and on the east coast of the United States, during the summer of 1880, by the U.S. coast survey steamer “Blake”, commander J.R. Bartlett, U.S. Bulletin of the Museum of Comparative Zoology at Harvard 10: 227–287. https://www.biodiversitylibrary.org/page/4211367

[B45] Mah CL McKnight DG Eagle MK Pawson DL Améziane N Vance DJ Baker AN Clark HES Davey N (2009) Phylum Echinodermata: sea stars, brittle stars, sea urchins, sea cucumbers, sea lilies. In: Gordon DP (Ed.) New Zealand inventory of biodiversity: 1.Kingdom Animalia: Radiata, Lophotrochozoa, Deuterostomia, 371–400.

[B46] Martynov A (2010) Reassessment of the classification of the Ophiuroidea (Echinodermata), based on morphological characters. I. General character evaluation and delineation of the families Ophiomyxidae and Ophiacanthidae. Zootaxa 2697: 1–54. 10.11646/zootaxa.2697.1.1

[B47] Matsumoto H (1911) About Japanese Euryalidae. Dobutsugaku Zasshi Tokyo. Zoological Magazine 23: 617–631. 10.4044/joma1889.23.258_631

[B48] Matsumoto H (1915) A new classification of the Ophiuroidea: with descriptions of new genera and species. Proceedings of the Academy of Natural Sciences of Philadelphia 128: 43–92.

[B49] Matsumoto H (1917) A monograph of Japanese Ophiuroidea, arranged according to a new classification. Journal of the College of Science, Imperial University, Tokyo 38: 1–408. https://www.biodiversitylibrary.org/page/7145928#page/5/mode/1up

[B50] McKnight DG (1989) Synoptic keys to the genera of Ophiuroidea. Zoology publications from Victoria University of Wellington 26: 1–44.

[B51] McKnight DG (2000) The Marine Fauna of New Zealand: Basket-stars and Snake-stars (Echinodermata: Ophiuroidea: Euryalinida). NIWA N Biodiversity Memoir, National Institute of Water and Atmospheric Research (NIWA), Wellington 115: 1–79.

[B52] McKnight DG (2003) *Asterostegus* (Echinodermata: Ophiuroidea) from the Cook Islands, South Pacific Ocean. Species Diversity 8: 385–389. 10.12782/specdiv.8.385

[B53] Mortensen T (1933) Echinoderms of South Africa (Asteroidea and Ophiuroidea) Papers from Dr. Th. Mortensen’s Pacific Expedition 1914–16. Videnskabelige Meddelelser fra Dansk naturhistorisk Forening 93 65: 215–400.

[B54] Müller J Troschel FH (1842) System der Asteriden.1. Asteriae. 2. Ophiuridae. Smithsonian, Vieweg: Braunschweig, [xxx+] 134 pp. [12 pls.] http://www.biodiversitylibrary.org/item/44159

[B55] Murakami S (1944) Report on the ophiurans from Ogasawara Islands and from off the Yeayama group, Nippon. Journal Department Agriculture Kyushu imperial University 7: 235–257. 10.5109/22600

[B56] O’Hara TD Hugall AF Thuy B Stöhr S Martynov AV (2017) Restructuring higher taxonomy using broad-scale phylogenomics: The living Ophiuroidea. Molecular Phylogenetics and Evolution 107: 415–430. 10.1016/j.ympev.2016.12.006 27940329

[B57] O’Hara TD Hugall AF Woolley SNC Bribiesca-Contreras G Bax NJ (2019) Contrasting processes drive ophiuroid phylodiversity across shallow and deep seafloors. Nature 565: 636–639. 10.1038/s41586-019-0886-z 30675065

[B58] OBIS (2021) Ocean Biodiversity Information System. Intergovernmental Oceanographic Commission of UNESCO. www.obis.org [accessed 15 August 2021] 10.18356/22202293-2021-1-10

[B59] Okanishi M Fujita T (2009) A New Species of *Asteroschema* (Echinodermata: Ophiuroidea: Asteroschematidae) from Southwestern Japan. Species Diversity 14: 115–129. 10.12782/specdiv.14.115

[B60] Okanishi M Fujita T (2013) Molecular phylogeny based on increased number of species and genes revealed more robust family-level systematics of the order Euryalida (Echinodermata: Ophiuroidea). Molecular Phylogenetics and Evolution 69: 566–580. 10.1016/j.ympev.2013.07.021 23906601

[B61] Okanishi M Fujita T (2014) A taxonomic review of the genus *Asterostegus* (Echinodermata: Ophiuroidea), with the description of a new species Masanori. Zoological Science 28: 148–157. 10.2108/zsj.28.148 21303207

[B62] Okanishi M Fujita T (2018) A taxonomic review of the genus *Astrodendrum* (Echinodermata, Ophiuroidea, Euryalida, Gorgonocephalidae) with description of a new species from Japan. Zootaxa 4392: 289–310. 10.11646/zootaxa.4392.2.4 29690406

[B63] Okanishi M O’Hara TD Fujita T (2011) Molecular phylogeny of the order Euryalida (Echinodermata: Ophiuroidea), based on mitochondrial and nuclear ribosomal genes. Molecular Phylogenetics and Evolution 61: 392–399. 10.1016/j.ympev.2011.07.003 21798356

[B64] Okanishi M Sentoku A Martynov A Fujita T (2018) A new cryptic species of *Asteronyx* Müller and Troschel, 1842 (Echinodermata: Ophiuroidea), based on molecular phylogeny and morphology, from off Pacific Coast of Japan. Zoologischer Anzeiger 274: 14–33. 10.1016/j.jcz.2018.03.001

[B65] Olbers JM Samyn Y Griffiths CL (2015) New or notable records of brittle stars (Echinodermata: Ophiuroidea) from South Africa. African Natural History 11: 83–116. 10.17159/2305-7963/2015/v11n1a3

[B66] Olbers JM Griffiths CL O’Hara TD Samyn Y (2019) Field guide to the brittle and basket stars (Echinodermata: Ophiuroidea) of South Africa. Abc Taxa 19: 1–354. http://www.abctaxa.be/volumes/volume_19_fieldguide-brittle-and-basket-stars

[B67] Pallas PS (1788) Marina varia nova et rariora. Nova Acta Academiae Scientiarum Imperialis Petropolitanea 2: 229–249. [plates 5–7] https://biodiversitylibrary.org/page/10095676

[B68] Parameswaran UV Jaleel AKU (2012) *Asteroschemasampadae* (Ophiuroidea: Asteroschematinae), A new deep-sea brittle star from the continental slope off the southern tip of India. Zootaxa 56: 47–56. 10.11646/zootaxa.3269.1.4

[B69] Paterson GLJ (1985) The deep-sea Ophiuroidea of the North Atlantic Ocean. Bulletin of the British Museum (Natural History) 49: 1–162. http://biodiversitylibrary.org/page/2273511

[B70] Pawson DL Vance DG Messing CG Solis-Marin FA Mah CL (2009) Echinodermata of the Gulf of Mexico. In: Felder DL DK Camp (Eds) Gulf of Mexico – Origins, Waters, and Biota.A & M University, Texas, 1177–1204.

[B71] Philippi A (1858) Beschreibungen einiger neuer Seesterne aus dem Meere von Chiloe. Archiv für Naturgeschichte 24: 264–268. http://biodiversitylibrary.org/page/13715200

[B72] Smirnov IS Piepenburg D Ahearn C Juterzenka KV (2014) Deep-sea fauna of European seas: An annotated species check-list of benthic invertebrates living deeper than 2000 m in the seas bordering Europe. Ophiuroidea. Invertebrate Zoology 11: 192–209. 10.15298/invertzool.11.1.18

[B73] Stöhr S O’Hara T Thuy B [Eds] (2021) The World Ophiuroidea Database. http://www.marinespecies.org/ophiuroidea [Accessed on 2021-09-07] 10.14284/358

[B74] Stöhr S (2011) New records and new species of Ophiuroidea (Echinodermata) from Lifou, Loyalty Islands, New Caledonia. Zootaxa 50: 1–50. 10.11646/zootaxa.3089.1.1

[B75] Stöhr S (2012) Ophiuroid (Echinodermata) systematics—where do we come from, where do we stand and where should we go? Zoosymposia 7: 147–162. 10.11646/zoosymposia.7.1.14

[B76] Stöhr S O’Hara TD (2021) Deep-sea Ophiuroidea (Echinodermata) from the Danish Galathea II Expedition, 1950–52, with taxonomic revisions. Zootaxa 4963: 505–529. 10.11646/zootaxa.4963.3.6 33903543

[B77] Stöhr S O’Hara TD Thuy B (2012) Global diversity of brittle stars (Echinodermata: Ophiuroidea). PLoS ONE 7: e31940. 10.1371/journal.pone.0031940 PMC3292557 22396744

[B78] Thompson JD Higgins DG Gibson TJ (1994) CLUSTALW: Improving the sensitivity of progressive multiple sequence alignment through sequence weighting, position-specific gap penalties and weight matrix choice. Nucleic Acids Research 22: 4673–4680. 10.1093/nar/22.22.4673 7984417 PMC308517

[B79] Turner RL Boucher JM O’Neill BO Becker NW (2021) Brittle stars with a bite: a new kind of pedicellaria in echinoderms. Zoomorphology 140: 505–525. 10.1007/s00435-021-00542-4

[B80] Verrill AE (1894) Descriptions of new species of starfishes and ophiurans, with a revision of certain species formerly described; mostly from the collections made by the United States Commission of Fish and Fisheries. Proceedings of the United States National Museum 17: 245–297. 10.5479/si.00963801.1000.245

[B81] Verrill AE (1899) Report on the Ophiuroidea collected by the Bahama expedition in 1893. Bulletin from the Laboratories of Natural History of the State University of Iowa 5: 1–86.

[B82] Ward RD Holmes BH O’Hara TD (2008) DNA barcoding discriminates echinoderm species. Molecular Ecology Resources 8: 1202–1211. 10.1111/j.1755-0998.2008.02332.x 21586007

